# Comparison of different promoters to improve AAV vector-mediated gene therapy for neuronopathic Gaucher disease

**DOI:** 10.1093/hmg/ddae081

**Published:** 2024-05-16

**Authors:** Giulia Massaro, Amy F Geard, Hemanth R Nelvagal, Katrina Gore, Nadine K Clemo, Simon N Waddington, Ahad A Rahim

**Affiliations:** UCL School of Pharmacy, University College London, 29-38 Brunswick Square, London, WC1N 1AX, United Kingdom; UCL School of Pharmacy, University College London, 29-38 Brunswick Square, London, WC1N 1AX, United Kingdom; Wits/SAMRC Antiviral Gene Therapy Research Unit, Faculty of Health Sciences, University of the Witwatersrand Medical, School, 7 York Road, Parktown 2193, South Africa; UCL School of Pharmacy, University College London, 29-38 Brunswick Square, London, WC1N 1AX, United Kingdom; Apollo Therapeutics, Stevenage Bioscience Catalyst, 50-60 Station Road, Cambridge, CB1 2JH, United Kingdom; Apollo Therapeutics, Stevenage Bioscience Catalyst, 50-60 Station Road, Cambridge, CB1 2JH, United Kingdom; Wits/SAMRC Antiviral Gene Therapy Research Unit, Faculty of Health Sciences, University of the Witwatersrand Medical, School, 7 York Road, Parktown 2193, South Africa; UCL EGA Institute for Women's Health, University College London, Medical School Building, 74 Huntley Street, London, WC1E 6AU, United Kingdom; UCL School of Pharmacy, University College London, 29-38 Brunswick Square, London, WC1N 1AX, United Kingdom

**Keywords:** gaucher disease, type 2 gaucher, GBA1, AAV9, neonatal gene therapy

## Abstract

Gaucher Disease (GD) is an inherited metabolic disorder caused by mutations in the *GBA1* gene. It can manifest with severe neurodegeneration and visceral pathology. The most acute neuronopathic form (nGD), for which there are no curative therapeutic options, is characterised by devastating neuropathology and death during infancy. In this study, we investigated the therapeutic benefit of systemically delivered AAV9 vectors expressing the human *GBA1* gene at two different doses comparing a neuronal-selective promoter with ubiquitous promoters. Our results highlight the importance of a careful evaluation of the promoter sequence used in gene delivery vectors, suggesting a neuron-targeted therapy leading to high levels of enzymatic activity in the brain but lower GCase expression in the viscera, might be the optimal therapeutic strategy for nGD.

## Graphical Abstract

**Graphical Abstract:**
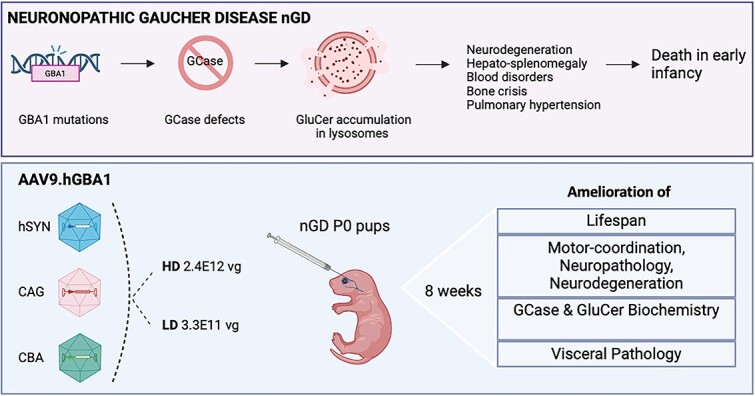

## Introduction

Gaucher Disease (GD) is the most common lysosomal disorder [1, 2], with estimated prevalence at 1:40000 to 1:150000 people worldwide [3]. GD is caused by mutations in the *GBA1* gene leading to defects in the lysosomal enzyme glucosylcerebrosidase (GCase), responsible for the cleavage of the β-glucosidic linkage of its substrates glucosylceramide (GluCer) [4]. GD is characterised by accumulation of GluCer, Lyso- and other sphingolipids like glucosylsphingosine (GluSph, Lyso-Gb1) in lysosomes of macrophages leading to disruption of the endo-lysosomal system, autophagy and other cellular pathways [5]. Engorged macrophages, known as Gaucher cells, are usually found in liver, spleen, lungs, bone marrow and brain of GD patients [3]. Historically, GD has been classified into three forms, based on the absence or presence of neurological manifestations, with type 1 being characterised by visceral symptoms and type 2 and 3 described as neuronopathic GD (nGD) [6]. Systemic manifestations vary, with the most common symptoms being hepatosplenomegaly, anaemia and thrombocytopenia, bone crisis with osteonecrosis of the joints, pulmonary hypertension, and less commonly, renal, and cardiac complications [1, 7]. While most type 1 and type 3 patients present visceral symptoms during childhood, some patients with milder GD display symptoms later in life. Type 2 is the most acute form of nGD, with accumulation of toxic biomolecules already in the perinatal period, onset of the first symptoms in the neonatal period and premature death by the age of 4 years [8]. Type 3 is the chronic form of nGD, characterised by slower progression and longer lifespan. In reality, GD exhibits a continuum of phenotypes with most patients showing some degree of neurological involvement [6, 9, 10]. Type 1 patients may develop subtle eye movement disorders or more severe neuropathy associated with traits of dementia, characterised by neuroinflammation with or without neuronal loss in the visual cortex and hippocampus [11]. GD has been linked to other neurodegenerative diseases such as Parkinson’s disease (PD) [12, 13]; mutations in the *GBA1* gene have been identified as a common risk factor in many PD patients. The heterogeneity of the symptoms, together with the unclear genotype/phenotype correlation, make early diagnosis challenging, often consigning patients and their families to a long and distressing journey.

Enzyme replacement therapy (ERT) and substrate reduction therapy (SRT) have been mainstay treatments for GD [14]. Three enzyme formulations are currently available, administered intravenously once every two weeks; and two SRTs for adult patients administered orally daily [14]. While ERT is safe and effective in reducing hepatosplenomegaly and ameliorate anaemia and thrombocytopenia, the recombinant enzyme does not cross the blood–brain barrier. Therefore it is not suitable for the treatment or management of the devastating neurological symptoms of nGD patients [15]. Other pharmacological therapies such as pharmacological chaperone therapy have been explored as part of the standard of care for GD patients, particularly for the paediatric population [16].

In the last decade, adeno-associated-virus (AAV)-mediated gene delivery has been proven to be effective and safe. Currently, AAV9 is the most widely used viral vector for pre-clinical studies and clinical trials for neurological disorders [17]. The success of AAV gene therapy for brain diseases in pre-clinical models is based on the ability of AAV9 to achieve a widespread transduction and transgene expression throughout the whole brain and spinal cord following a single systemic dministration [18–20]. In addition, administration of AAV9 in combination with different promoter sequences in the perinatal period has been shown to result in preferential transduction of neurons over astrocytes in animal models [21–23]. Evidence of clinical safety and efficacy of intravenous AAV9 gene therapy has been demonstrated in the clinical trial for paediatric Spinal Muscular Atrophy type 1 (SMA1) [24, 25], with consequent FDA approval of onasemnogene abeparvovec in 2019 as the first gene therapy drug for a neurological disorder.

Proof-of-concept using AAV-based gene therapy as a successful treatment option for nGD has been previously demonstrated, where early intervention in the fetal [26] or perinatal period [27] rescued the severe neurodegeneration of a nGD mouse model. The K14-lnl/lnl type 2 nGD model [28] exhibits acute neurovisceral pathology, leading to premature death at 12–14 days of age. While the severe neuropathology was significantly improved using a strong neuronal promoter [27] resulting in prolonged life span, the low levels of circulating enzyme were not sufficient to significantly ameliorate the visceral pathology in all organs. Therefore, there is a need to determine the ideal dosing as well as choice of promoter to achieve the optimal therapeutic outcome for further translation into humans. In this study, our aim was to develop a unified therapeutic strategy for the Gaucher population, improving both the visceral and the neurological symptoms, and evaluate the effects of sustained supraphysiological body-wide GCase expression. We have therefore sought to compare the therapeutic effects of the previously established neuronal specific vector versus two novel AAV9 constructs, where the expression of the human *GBA1* gene is driven by the ubiquitous chicken β-actin promoter in the CBA and CAG sequences, with the CAG promoter having recently been utilized in the successful translation of onasemnogene abeparvovec and is currently utilised in phase 1/2 trials for type 1, type 2 and PD-GBA patients (clinicaltrials.gov: NCT05487599, NCT04411654, NCT04411654).

Our results show for the first time that high doses of systemically delivered gene therapy across all three promoters resulted in extended life span, normalisation of neuropathological markers and increased enzymatic activity in brain and visceral organs. However, supraphysiological glucosylcerebrosidase (GCase) levels in the viscera resulting from the ubiquitous promoters was indicative of a possible deleterious effect in these organs. Taken together, our data suggest that the strong neuronal promoter appears to have the greatest benefit across the CNS and viscera overall, and that the careful evaluation of dosing and choice of promoter are crucial to successful and safe translation of gene therapy for nGD.

## Results

### Gene therapy rescues the nGD mouse model in a dose-dependent fashion, with the synapsin promoter outperforming the ubiquitous promoters at both a high and low dose

The *pAAV.hSynI.hGBA1*, *pAAV.CAG.hGBA1* and *pAAV.CBA.hGBA1* plasmid constructs (Fig. S1A) were first tested *in vitro* to confirm *GBA1* expression. All three constructs led to increased levels of GCase activity in HEK-293 transfected cells (Fig. S1B), with *CAG* and *CBA* driving significantly higher expression compared to *hSynI* in the cell lysates. In addition, higher levels of GCase were secreted to the supernatant in cells transfected with *pAAV.CAG.hGBA1* and *pAAV.CBA.hGBA1* (Fig. S1C), confirming these ubiquitous sequences promoted high expression in non-neuronal cells.

**Figure 1:**
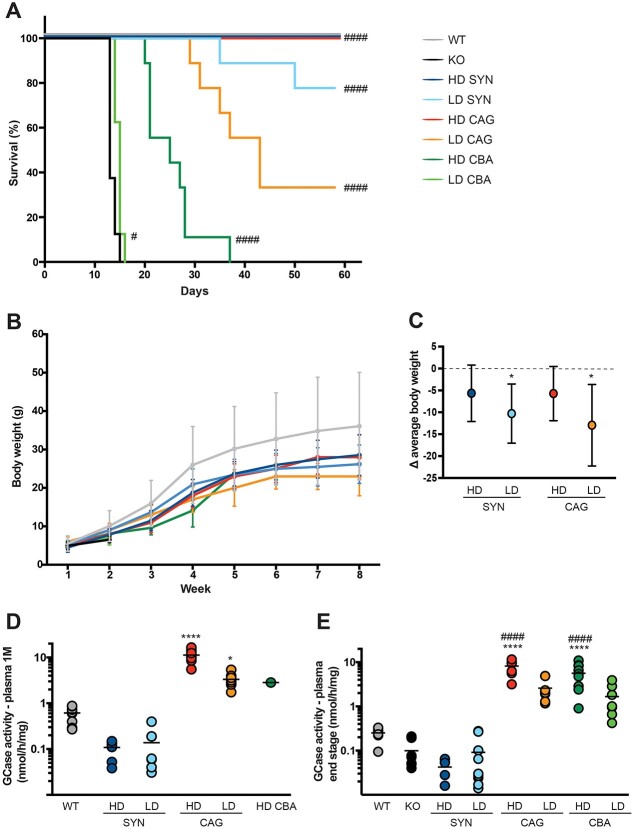
Gene therapy rescues the K14-lnl/lnl model in a dose-dependent fashion. (A) Kaplan–Meier survival curve. Data expressed as percentage of survival. (B) body weight expressed as average weight of each cohort/week. (C) difference in average body weight compared to WT (dotted line) at 8 weeks. Data expressed as average weight ± 95% CI. (D) GCase enzymatic activity in plasma 1 month post-administration. Data presented as single data points and mean. (E) GCase enzymatic activity in plasma at end-stage collection point. Data presented as single data points and mean. ^*^indicates statistically significant difference between the experimental group and WT controls; # indicates statistically significant difference between the experimental group and untreated KO controls. *P* values and statistical tests are reported in Table S3.

The plasmid constructs were then used to produce AAV9 viral vectors. K14-lnl/lnl knock out (KO) mice were injected intravenously with either high (HD: 2.4×10^12^ vg) or low dose (LD: 3.3×10^11^ vg) of each of the three AAV9 vectors: SYN.hGBA, CAG.hGBA and CBA.hGBA. The same high dose was previously used to deliver the SYN.hGBA vector to KO pups [27] with consequent extension of the life span; the low dose was chosen to prove dose-dependent efficacy of 10-fold diluted vectors with the aim of maintaining efficacy while reducing the possible risks of immunogenicity associated to high doses of vector [29]. Untreated KOs and wild-type (WT) animals were used as littermate controls. Mice were kept for 8 weeks, or collected earlier if they reached their humane end point (Fig. 1A). Consistent with previous studies, the untreated KOs did not survive 14 days. Treatment with HD SYN.hGBA and HD CAG.hGBA resulted in 100% survival of injected KOs to 8 weeks. While 7/9 mice treated with LD SYN.hGBA reached 8 weeks (average survival = 53.8 days), only 3/9 animals injected with LD CAG.hGBA survived to the end of the experiment (average survival = 43 days). Nevertheless, both LD SYN.hGBA and LD CAG.hGBA led to significant increase in lifespan compared to untreated KOs. Treatment with the CBA.hGBA vector at high dose resulted in average survival of 25.3 days, while KOs injected with LD CBA.hGBA did not survive over 2 weeks (average survival = 14.7 days), comparable to untreated KOs.

Body weight was monitored weekly (Fig. 1B). Treated mice did not lose weight, although most cohorts did not reach WT levels, with LD SYN.hGBA and LD CAG.hGBA 8-week-old mice being on average significantly smaller than 8-week-old WTs (Fig. 1C). At collection point, 8-week-old HD SYN.hGBA and HD CAG.hGBA treated KOs were smaller, although not significantly, than WT age-matched controls.

Circulating GCase enzymatic activity was measured in plasma at 4 weeks post-treatment (Fig. 1D) and at collection end-stage (Fig. 1E). Circulating levels of GCase were significantly higher in animals treated with the vectors carrying the ubiquitous vectors CAG.hGBA and CBA.hGBA, with treated mice expressing supraphysiological levels at both 1 month post-injection and end-point. In particular, treatment with CAG.hGBA resulted in significant and sustained high GCase levels at 8 weeks post-injection. Despite significant extension in life span, treatment with the neuronal specific vector SYN.hGBA did not result in high levels of circulating enzyme at any of the time points.

### Optimal combined promoter selection and dosing prevents neuropathology in the brain

**Figure 2:**
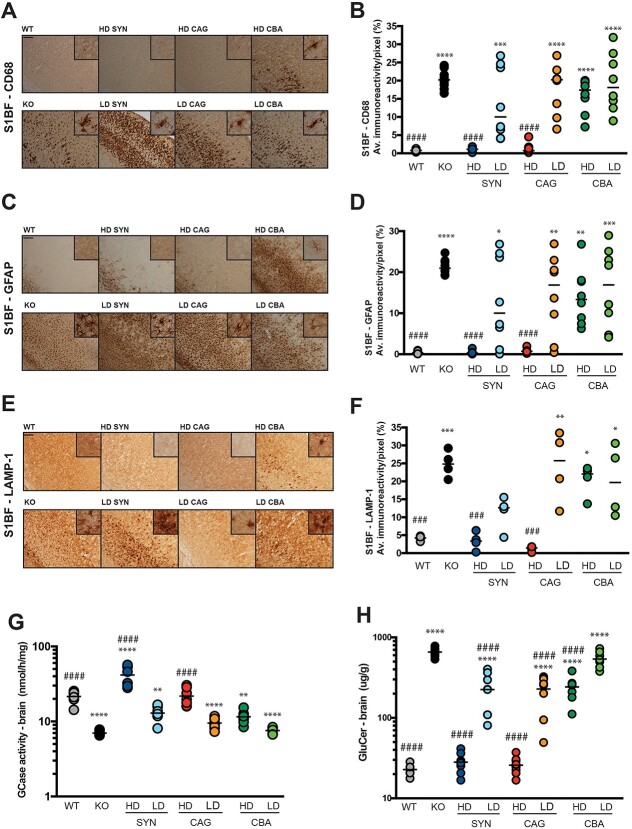
Gene therapy ameliorates neuropathology and reduces substrate accumulation in the brain. (A) representative images of brain sections (cortical region S1BF) stained for the macrophagic marker CD68. Scale bar: 100 μm; high magnification inserts: 60 μm. (B) quantification of immunoreactivity of brain sections stained for CD68. (C) representative images of brain sections (cortical region S1BF) stained for the astrocytic marker GFAP. Scale bar: 100 μm; high magnification inserts: 60 μm. (D) quantification of immunoreactivity of brain sections stained for GFAP. (E) representative images of brain sections (cortical region S1BF) stained for the lysosomal marker LAMP1. Scale bar: 100 μm; high magnification inserts: 60 μm. (F) quantification of immunoreactivity of brain sections stained for LAMP1. (G) GCase enzymatic activity in brain homogenates. (H) substrate accumulation (GluCer isoform C18:0) in brain homogenates. All data are presented as single data points and mean. ^*^indicates statistically significant difference between the experimental group and WT controls; # indicates statistically significant difference between the experimental group and untreated KO controls. *p* values and statistical tests are reported in Table S4.

Extensive histopathological analysis was conducted on brain tissue to evaluate the effects of the therapies on the severe neuropathology that characterises the K14-lnl/lnl nGD mouse model. Three brain regions, the most affected in the mouse model and representative of the human disease course, were examined: the somatosensory barrel field cortex S1BF (Fig. 2A-F), the ventral posteromedial and posterolateral nuclei VPM/VPL in the thalamus (Fig. S2), and the gigantocellular nuclei Gi in the brain stem (Fig. S2). In the cortex, KO brains are characterised by extensive microglia activation (Fig. 2A-B), astrogliosis (Fig. 2C-D), and accumulation of enlarged lysosomes (Fig. 2E-F). Treatments with either HD SYN.hGBA or HD CAG.hGBA completely normalised CD68 (Fig. 2B), GFAP (Fig. 2D), and LAMP1 (Fig. 2F) markers to WT levels. Gene therapy had an effect on the pathology in a dose-dependent fashion, where the same SYN and CAG vectors administered at a lower dose did not completely rescue the severe neuropathology of the model. Although some animals showed improvement in microglia activation, astrogliosis and lysosomal accumulation, the effect was variable and the pathology in some mice of the LD treated cohorts was similar to untreated KO brains. Again, treatment with the CBA.hGBA vector led to minimal or no amelioration of the neuropathology in the cortex of injected animals, regardless of the dose (Fig. 2A-F). Although LD treatments did not completely reverse the neuropathology, one must consider that most of the administered mice were older than the 14-day-old untreated KO controls. This suggests that the rate at which the pathology progressed was at least partially reduced, delaying the onset of the neuropathology.

A similar pattern was observed when the other brain regions were analysed (Fig. S2). While SYN.hGBA and CAG.hGBA completely cleared the neuropathological markers in the thalamus and brain stem when administered at high dose, low dose treatments did not result in extensive and significant improvements compared to KO. However, the LD SYN.hGBA cohort showed greater effects, with normalisation of the CD68 marker in the brain stem (Fig. S2B and H), and LAMP1 index in the thalamus (Fig. S2E and K) and brain stem (Fig. S2F and L) to WT levels. Similar results were observed in the cerebellum, with HD SYN.hGBA and HD CAG.hGBA restoring all neuropathological markers to WT levels (data not shown). None of the neuropathological indexes were restored following CBA.hGBA administration.

Overall, treatment with HD SYN.hGBA and HD CAG.hGBA resulted in normalisation of various pathological phenotypes throughout the analysed brain regions. LD SYN.hGBA ameliorated lysosomal accumulation, performing significantly better than LD CAG.hGBA in all the analysed brain regions (Fig. 2E-F, Fig. S2K-L).

Histopathology was complemented with a biochemical analysis evaluating GCase enzymatic activity (Fig. 2G) and GluCer substrate levels (isoform C18:0 Fig. 2H). A full mass spectrometry panel C16:0-C24:0 in whole brain homogenates is shown in (Fig. S3A). Animals treated with either HD SYN.hGBA or HD CAG.hGBA showed normalised levels of enzyme in the brain, with the neuronal vector leading to supraphysiological levels of GCase activity. Low doses and the CBA vector did not significantly improve or normalise enzyme activity. The reduction of the accumulating substrate was normalised to WT levels in the HD SYN.hGBA and HD CAG.hGBA cohorts. Partial amelioration was observed in the other groups, where GluCer accumulation was significantly lower than the untreated KO controls yet not reaching physiological WT level.

### The synapsin promoter is optimal in preventing neuronal loss in 8-weeks-old mice and restoring locomotor function

**Figure 3:**
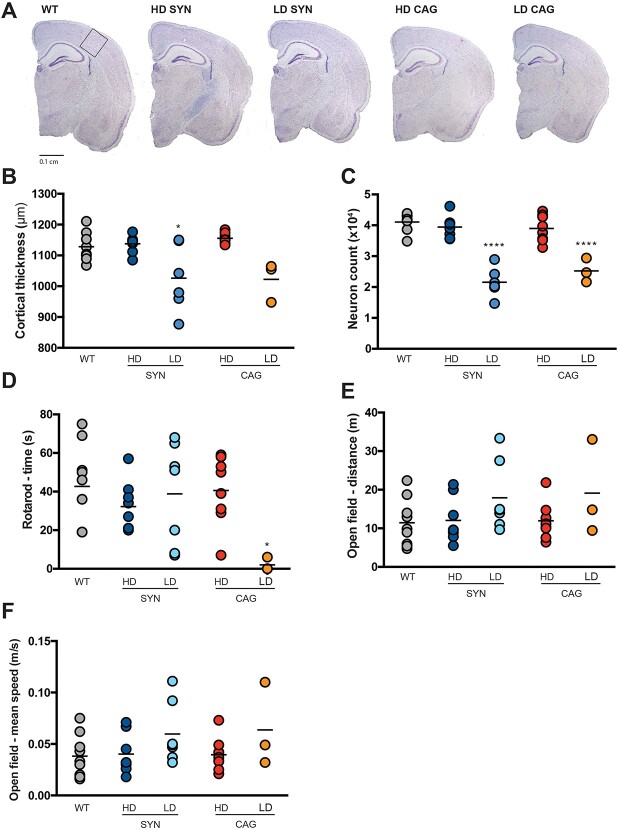
High dose gene therapy rescues neurodegeneration in the cortex and normalises behavioural indexes at 8 weeks post-administration. (A) representative images of brain sections stained with Nissl. Insert represents the S1BF cortical region. Scale bar: 0.1 cm. (B) quantification of cortical thickness in 8-week-old mice. (C) neuron count in the S1BF cortical region in 8-week-old mice. (D) time spent on the accelerating rotarod. (E) distance travelled in the open field chamber in a 5-minute time period. (F) mean speed of travel during open field analysis. All data are presented as single data points and mean. ^*^indicates statistically significant difference between the experimental group and WT controls. *p* values and statistical tests are reported in Table S5.

Brain sections were stained with Nissl to evaluate tissue morphology and perform a stereological analysis on 8-week-old mice. From an initial observation of the sections (Fig. 3A), treated brains did not show evident disruption of the tissue architecture, atrophy or ventriculomegaly. However, following estimation of the dimension of the S1BF cortical region, it appeared that the LD SYN.hGBA and LD CAG.hGBA cohorts were characterised by cortical atrophy compared to the HD treated animals and WT controls (Fig. 3B). Similar results were obtained from stereological count of neurons in the S1BF region (Fig. 3C). Mice treated with low dose gene therapy showed significant reduction of neuronal cells; whereas treatment with HD SYN.hGBA and HD CAG.hGBA led to complete normalisation of neuron count to WT levels.

8-week-old mice underwent behavioural analyses before collection, to assess whether gene therapy improved locomotor function in treated mice. Time spent on the accelerating rotarod (Fig. 3D), distance travelled in the open field chamber (Fig. 3E) and average speed (Fig. 3F) were recorded. Animals treated with the SYN.hGBA vector, either at high or low dose, did not show significant deterioration in any of the parameters analysed. Similarly, mice administered with the CAG.hGBA vector did not display evident motor coordination deficits, except for the LD CAG.hGBA cohort showing a reduced ability of performing the rotarod test (Fig. 3D). Again, the therapeutic outcomes of the treatments were dose-dependent, with high-dose treated mice performing marginally better than their counterparts treated with the same vector at low doses. Interestingly, mice treated with LD SYN.hGBA and LD CAG.hGBA showed mild—albeit not significant signs of hyperactivity (higher speed and longer distance travelled in the open field chamber).

### The CAG promoter is optimal in mediating GCase enzymatic activity and improving substrate accumulation in visceral organs

**Figure 4:**
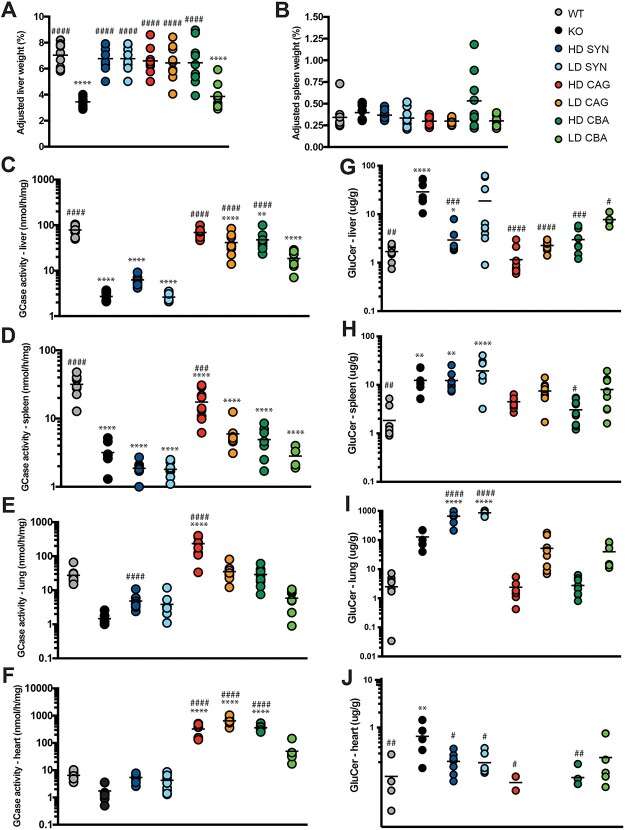
Biochemical analysis reveals significant increase in GCase enzymatic activity following ubiquitous gene therapy. (A) liver weight expressed as percentage of total body weight. (B) spleen weight expressed as percentage of total body weight. (C) GCase enzymatic activity in liver homogenates. (D) GCase enzymatic activity in spleen homogenates. (E) GCase enzymatic activity in lung homogenates. (F) GCase enzymatic activity in heart homogenates. (G) substrate accumulation (GluCer isoform C24:0) in liver. (H) substrate accumulation (GluCer isoform C24:0) in spleen. (I) substrate accumulation (GluCer isoform C24:0) in lung. (J) substrate accumulation (GluCer isoform C24:0) in liver. All data are presented as single data points and mean. ^*^indicates statistically significant difference between the experimental group and WT controls; # indicates statistically significant difference between the experimental group and untreated KO controls. *P* values and statistical tests are reported in Table S6.

One of the main phenotypic manifestations of GD is hepato-splenomegaly. The neuropathology of the K14-lnl/lnl type 2 nGD mouse model is severe and the animals do not survive long enough for the visceral pathology to progress and display an evident enlargement of the liver and spleen. In our previous work^29^ we have demonstrated that gene therapy administered to the brain of KO mice progressively develop visceral pathology, therefore we wanted to evaluate whether systemic treatment would influence the size of the liver (Fig. 4A) and spleen (Fig. 4B). The organ weight was normalised to body weight. Untreated KOs and the LD CBA.hGBA cohort showed significantly small ratio of liver to body weight. This is not unexpected, since KOs have averagely lower weight than the treated littermates, and in animals collected within 2 weeks the liver has not gone through the exponential growth up to post-natal week 8. All other cohorts did not develop enlargement of either liver or spleen at time of collection.

Liver (Fig. 4C), spleen (Fig. 4D), lung (Fig. 4E) and heart (Fig. 4F) were collected and GCase enzymatic activity was measured. Overall, the ubiquitous vectors CAG.hGBA and CBA.hGBA promoted higher levels of enzyme compared to the neuronal vector SYN.hGBA in all analysed tissues. Although not always significantly, gene therapy with CAG.hGBA and CBA.hGBA vectors resulted in increased GCase activity compared to untreated KOs in all tissues. In particular, following treatment with HD CAG.hGBA GCase activity was normalised to WT levels in the liver and supraphysiological levels in the lung (Fig. 4E) and the heart (Fig. 4F). Supraphysiological levels of GCase activity were also reached in the heart following treatment with either LD CAG.hGBA or LD CBA.hGBA (Fig. 4F). Similarly to what was observed in the plasma, administration of the SYN.hGBA vector did not result in significantly increased GCase activity compared to untreated KOs in any of the analysed tissue, with the exception of the HD SYN.hGBA cohort which showed higher enzymatic activity in the lung compared to untreated controls (Fig. 4E).

The same organs were also analysed via mass spectrometry to evaluate the effect of systemic gene therapy on the accumulation of the main toxic substrate glucosylceramide (GluCer isoform C24:0 Fig. 4G-L. Full mass spectrometry panel C16:0-C24:0 Fig. S3B-E). An increase in GCase expression and activity should correlate to reduced GluCer accumulation. Indeed, mass spectrometry results mirrored previous findings, with CAG.hGBA- and CBA.hGBA treated mice showing significantly reduced GluCer accumulation compared to KOs. Substrate levels were normalised to WT range. Again, treatment with the neuronal vector SYN.hGBA did not decrease substrate accumulation in the spleen (Fig. 4H) and lungs (Fig. 4I) of treated mice, confirming GCase enzymatic activity was not fully restored in some of the visceral tissues. Mice treated with HD SYN.hGBA showed reduced GluCer accumulation in the liver compared to KOs, however the substrate was not completely normalised to WT levels (Fig. 4G). Both HD and LD SYN.hGBA decreased GluCer accumulation in the heart to WT levels (Fig. 4J). Interestingly, while enzymatic activity in the lung was comparable to WT (Fig. 4E), Accumulation of GluCer was significantly increased in mice treated with either dose of the SYN.hGBA vector.

### High dose SYN.hGBA is efficacious in normalising pathological markers in the visceral organs compared to ubiquitous promoters

**Figure 5:**
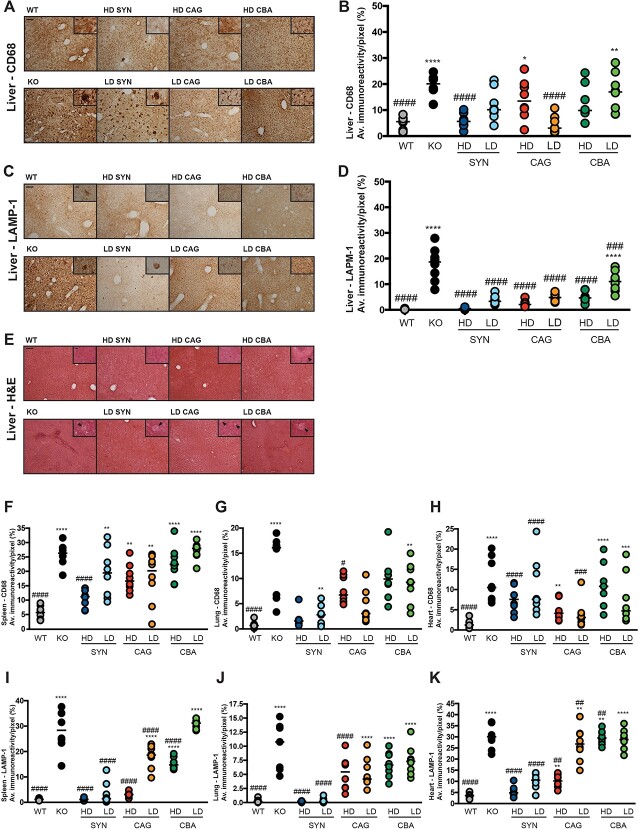
SYN.hGBA ameliorates pathological markers in the viscera of treated mice, while treatment with the ubiquitous vectors leads to increased inflammation. (A) representative images of liver sections stained for the macrophagic marker CD68. Scale bar: 100 μm; high magnification inserts: 60 μm. (B) quantification of immunoreactivity of liver sections stained for CD68. (C) representative images of liver sections stained for the lysosomal marker LAMP1. Scale bar: 100 μm; high magnification inserts: 60 μm. (D) quantification of immunoreactivity of liver sections stained for LAMP1. (E) representative images of liver sections stained with H&E. Arrows indicates Gaucher cells. Scale bar: 100 μm; high magnification inserts: 60 μm. (F) quantification of immunoreactivity of spleen sections stained for CD68. G quantification of immunoreactivity of lung sections stained for CD68. (H) quantification of immunoreactivity of heart sections stained for CD68. (I) quantification of immunoreactivity of spleen sections stained for LAMP1. (J) quantification of immunoreactivity of lung sections stained for LAMP1. (K) quantification of immunoreactivity of heart sections stained for LAMP1. All data are presented as single data points and mean. ^*^indicates statistically significant difference between the experimental group and WT controls; # indicates statistically significant difference between the experimental group and untreated KO controls. *P* values and statistical tests are reported in Table S7.

The biochemical analyses on the most affected visceral organs were complemented with a pathology study using the pan-macrophage marker CD68 to evaluate the effect of the therapies on the accumulation of enlarged macrophages, the LAMP1 marker as an indicator of expansion of the endo-lysosomal system, and hematoxylin/eosin (H&E) staining to evaluate cell and tissue architecture, as well as the occurrence of Gaucher cells (Fig. 5 and Fig. S4). As previously shown [26–28], the K14-lnl/lnl model is characterised by accumulation of engorged macrophages and swollen lysosomes in the liver (Fig. 5A and C), spleen (Fig. S4A and D), lung (Fig. S4B and E) and heart tissues (Fig. S4C and F). Systemic treatment with HD SYN.hGBA and LD CAG.hGBA significantly reduced macrophage accumulation in the liver (Fig. 5B) to WT levels, while the CBA.hGBA vector did not effectively clear the CD68 pathology. The treatments displayed a dose-dependent trend (vector copy number analysis available in Fig. S5), with higher doses being more effective in clearing enlarged macrophages in the liver tissue. Unexpectedly, HD CAG.hGBA did not have a significant effect in normalising macrophage accumulation in the liver. However, the therapy showed variable outcomes with some mice displaying a significant reduction in CD68 pathology. The variation in these results was of particular interest, given the fact that all treated mice showed similar levels of elevated GCase activity in the liver (Fig. 4C). In addition, the SYN.hGBA vector was more efficient in preventing macrophagic inflammation than CAG.hGBA, despite GCase activity (Fig. 4C) and GluCer build-up (Fig. 4G) not being normalised to WT levels.

Similarly to macrophages, the endo-lysosomal system is severely affected in KO mice, with significant enlargement of the organelles in the liver (Fig. 5C). All three vectors had a therapeutic effect on lysosome accumulation (Fig. 5D), whether administered at a high or low dose. While mice treated with LD CBA.hGBA showed some residual LAMP1 staining, albeit not to KO level, the LAMP1 levels were completely normalised to WT in all the other cohorts.

Further analysis of liver tissue stained with H&E to assess tissue architecture (Fig. 5E) showed engorged macrophages (‘Gaucher cells’) in the KO organs, where the integrity of the tissue was disrupted. Liver sections from mice treated with HD SYN.hGBA and HD CAG.hGBA did not show any Gaucher cells and the tissue architecture was maintained. Some sparse Gaucher cells were identified in the LD SYN.hGBA and LD CAG.hGBA tissue. Liver tissue from mice injected with the CBA.hGBA vector presented numerous enlarged macrophages, with severe disruption of tissue integrity in the low dose treated group.

Spleen, lung and heart tissue was also stained for CD68 (Fig. S4A-C) and LAMP1 (Fig. S4D-F). Enlarged macrophages and enlarged lysosomes characterised the KO tissue, recapitulating the pathological features observed in GD patients. Quantification of immunoreactivity (Fig. 5F-K) showed that overall treatment with either SYN.hGBA or CAG.hGBA normalised or partially reduced macrophage and lysosome accumulation, with a dose-dependent effect. High doses were on average more effective in clearing the pathology compared to the low dose treatments. Interestingly, the neuronal SYN.hGBA vector had a stronger therapeutic effect on the lung compared to the ubiquitous vector CAG.hGBA, with lower levels of both CD68 (Fig. 5G) and LAMP1 (Fig. 5J) staining that was mostly normalised to WT levels. Quantification of the CD68 staining confirmed both HD and LD CBA.hGBA did not ameliorate the pathology, showing levels of macrophagic activation similar to untreated KOs in the spleen (Fig. 5F), lung (Fig. 5G) and heart (Fig. 5H). Analogous results were reported for LAMP1 staining, with significantly increased endo-lysosomal system expansion to KO levels (Fig. 5I-K).

H&E staining (Fig. S4G-I) confirmed the presence of engorged Gaucher cells, disruption in the normal tissue architecture in the KO tissue. Again, treatment with HD SYN.hGBA and HD CAG.hGBA resulted in almost complete clearance of Gaucher cells in spleen (Fig. S4G), lung (Fig. S4H) and heart (Fig. S4I). Administration of the two vectors at a lower dose ameliorated the pathology only to a limited extent and mildly improved tissue integrity, particularly in the white pulp of the spleen (Fig. S4G). Organs from mice treated with the CBA.hGBA vector displayed features similar to the KO tissue, with enlarged Gaucher cells and disrupted tissue architecture.

The histopathological assessment, coupled with the biochemical analyses, uncovered some interesting and unforeseen findings. Overall, the SYN.hGBA vector outperformed, or was at least as efficient as the ubiquitous vectors in clearing CD68-positive cells and reducing lysosome accumulation. This was rather unexpected, since the GCase enzymatic activity was significantly lower in the blood (Fig. 1D-E) and all visceral organs (Fig. 4). In comparison, mice treated with HD CAG.hGBA displayed elevated macrophage activation in the heart, despite having supraphysiological GCase activity levels leading to normalisation of substrate accumulation.

## Discussion

GD is a prime candidate for gene therapy due to its well-characterised gene defect and pathophysiology, the GCase enzyme being secreted which allows for cross-correction and the relatively large patient population. We have previously demonstrated the feasibility and efficacy of delivering the human *GBA1* gene to a mouse model of severe nGD, either to fetuses or neonates, using AAV9 viral vectors [26, 27]. Several gene therapy programs for GD are underway, particularly after a link between GD and Parkinson’s was established. Three phase 1/2 clinical trials using an AAV9.CAG vector to deliver *GBA1* to type 1 (clinicaltrials.gov: NCT05487599), type 2 (clinicaltrials.gov: NCT04411654), and PD patients with *GBA1* mutations (clinicaltrials.gov: NCT04127578) are currently ongoing. While the vector will be administered systemically to type 1 GD patients, gene therapy will be delivered to the cisterna magna (CM) to target the brain for either type 2 GD or PD patients. Pre-clinical studies in large animals have demonstrated that delivery of AAV vectors via this route is efficient in achieving widespread expression [30–32]. There is, however, high risk of off-target injections into the medulla with severe injury and associated complications [33]. In addition, our previous findings suggested that brain-directed gene delivery can rescue the severe neurodegeneration, but pathology still develops over time in the viscera and bones. Therefore, treated nGD patients might encounter debilitating systemic symptoms that could affect their quality of life and would have to be managed with concomitant administration of other therapies, such as ERT, in the long term.

The choice of using the *CAG* promoter sequence to achieve a full-body gene expression is clinically relevant, as it is included in onasemnogene abeparvovec [24] and is currently used in several other clinical trials. The rationale of using a ubiquitous strong promoter relies on the idea of a single systemic delivery of gene therapy, which could target both the visceral pathology and the neuropathology in all GD patients’ populations and mediate as much secretion of GCase into the extracellular space and blood plasma as possible to enhance efficacy. Interestingly, our findings are similar to the data reported in the FDA Biologics License Application for onasemnogene abeparvovec, where elevated doses of an AAV9.CAG vector can lead to adverse reaction in the heart. In our study, mice treated with HD CAG.hGBA presented supraphysiological GCase activity levels in the heart with consequent normalisation of GluCer accumulation. However, histological analysis showed increased macrophage activation in the tissue. Similar results were observed in other organs and also in mice treated with HD CBA.hGBA, but to a lesser extent in some of the organs of the mice treated with the SYN.hGBA vector, suggesting that this effect does not directly correlate with administration of the vector at a high dose but might be caused by increased GCase expression and/or activity. Administration of CAG.hGBA led to almost 100-fold increase in enzyme activity in the heart compared to WT, either in the HD or LD cohort. With such high levels of GCase activity, complete normalisation of the pathological markers was expected. Interestingly, this was not the case. We hypothesise that high and sustained levels of GCase protein can elicit CD68 inflammatory response. However, the mechanisms of these effects are not clear and further studies are necessary to define the nature of these inflammatory effects, and whether this could be attributed to a more generalised response to high dose AAV treatment. In addition, the quantification of vector genomes in the heart tissue showed a clear dose-dependent difference between the HD and LD groups. However, the measured enzymatic activity in the two cohorts was comparable. We could speculate the GCase enzymatic capacity of the cell becomes saturated once the threshold of physiological *GBA1* expression is surpassed. Additional studies administering the vector at a broader range of doses would be required to further investigate this hypothesis, particularly considering that previous studies injecting the SYN.hGBA vector at high doses to wild-type mice [27] have not shown indication of inflammation or macrophage activation.

Working with the K14-lnl/lnl mouse model can be extremely challenging, as the animals reach their humane endpoint within 2 weeks, and reversing the severe neuropathology in such a brief time window requires high levels of GCase expression. In our study we administered gene therapy at 3.3×10^14^ vg/kg (LD) and 2.5×10^15^ vg/kg (HD). To put these numbers into perspective, the HD cohorts received a dose 20-times higher than SMA patients (1.1×10^14^ vg/kg). Ours is a proof-of-concept investigation where we set out to demonstrate the feasibility of using a ubiquitous vector for the treatment of nGD. Different doses will be required to be tested in order to lower the vector load and reduce adverse effects, while maintaining the positive therapeutic outcome in the brain. In the light of recent clinical data, such as the reports describing adverse events of thrombotic microangiopathy in patients treated with AAV9 gene therapies for Duchenne muscular dystrophy [34] and spinal muscular atrophy [35], careful consideration must be given to dosing of viral vectors. Therefore, a thorough investigation of possible adverse effects must be conducted prior to administration of high doses of AAV vectors, performing comprehensive toxicology and dose-escalation studies. In addition, the possibility of lowering the dose, possibly using a different design or vector serotype, should be further explored in the light of previous gene transfer studies conducted on different murine models of Gaucher, where modest levels of expression promoted both systemic [36] and neurological [37] therapeutic effects.

Treatment of the K14-lnl/lnl model, with a view to develop a unified therapeutic strategy for all GD patients, requires a delicate balance between efficient clearance of the brain pathology and sufficient GCase expression in the viscera. Administration of the neuronal SYN.hGBA vector resulted in low circulating GCase levels and enzymatic activity in the viscera. Yet, all animals treated with HD and 7/9 mice in the LD cohort reached the end of the experiment at 8 weeks. This was not unexpected, considering the model is primarily affected by severe neurodegeneration and neuroinflammation. Macrophage and lysosome accumulation in the viscera was still present, indicating that the SYN.hGBA vector could be an excellent candidate for a brain-direct therapy but it might not be ideal for the treatment of type 1 patients. While a ubiquitously expressed vector is potentially a good therapeutic option to deliver the gene of interest to as many organs as possible, the effects of CAG.hGBA in the viscera opens a question about tissue and cell type specificity. Indeed, it might not be beneficial expressing *GBA1* at such high levels in all tissues, particularly in cells that normally do not produce or produce low concentrations of GCase.

This study confirms and adds further evidence in support gene therapy as a feasible therapeutic option for GD patients. Developing one single product (i.e: AAV9.CAG.hGBA) for different indications is a strategic approach that could undeniably be beneficial to the GD population, whether the treatment is directed to type 1, nGD or GD-related PD patients. Nonetheless, we must consider cautiously what vector, promoter sequence, dose, timing and route of administration would be the most suitable and safest for the patients, particularly for type 2 GD where children would have to be treated with high doses in early infancy. We have previously suggested that *in utero* gene therapy could be considered for type 2 cases, if associated with early and reliable diagnosis [38, 39]. It has not been established yet if treatment at the fetal stage could completely prevent the severe neuropathology, or whether an early intervention would only slow down the neurodegenerative process leading to chronic neurological disability later in life. Nevertheless, a recent landmark study describes safe and efficacious in utero enzyme replacement in Infantile-Onset Pompe’s Disease which provides supporting evidence that in utero intervention in lysosomal storage diseases may be beneficial [40].

## Materials and methods

### A‌AV plasmids and vectors production

The *cis*-plasmids *pAAV.hSynI.hGBA1*, *pAAV.CAG.hGBA1* and *pAAV.CBA.hGBA1* were cloned and *GBA1* expression was tested on HEK-293 cells. Cells were grown in Dulbecco’s Modified Eagle Medium DMEM GlutaMax (ThermoFisher) with 10% heat-inactivated Fetal Bovine Serum FBS (Gibco, ThermoFisher) and 1% Pen/Strep antibiotic (Gibco, ThermoFisher) in standard conditions of 37°C and 5% CO_2_. 250 000 cell/6-well plate were transfected with a mix containing 1 μg DNA, 3.5 mg/ml polyethylenimine PEI (Polyscience) and 500 μl Opti-MEM (Gibco, ThermoFisher) per each well. Each plasmid and untransfected controls were tested in 3 technical replicates.

Single-stranded AAV9 vectors were produced by Vector Biolabs (Malvern, PA). Endotoxin-free plasmid preparations were provided to Vector Biolabs. AAV vectors were produced by the standard triple plasmid transfection method, purified by 2 cycles of density gradient ultracentrifugation with CsCl and resuspended in phosphate-buffered saline (PBS). Each stock preparation was diluted in PBS to 6×10^13^ vg/ml and 8.25×10^12^ vg/ml.

### Animal welfare and colony maintenance

All listed procedures were approved by the UK Home Office for the conduct of regulated procedures under license (Animal Scientific Procedure Act, 1986) and by the University College London Animal Welfare and Ethical Review Board (AWERB). The Animal Research Reporting of *In Vivo* Experiments (ARRIVE) guidelines from the National Centre for the Replacement Refinement and Reduction of Animals in Research were followed. A loss of 15% body weight or signs of paralysis, spasticity, or unconsciousness for more than 4 h, were set as a humane endpoint. Animals were maintained in individually ventilated cages, on a 12 h light/dark cycle, with access to water and food *ad libitum*. The mouse colony was maintained as heterozygous and genotyped at birth as previously reported^30^. The animals were time-mated to synchronise birth of enough litters on the same day each week. The experimental cohorts were monitored for neurological symptoms and weighted weekly. The sex of the animals was confirmed and recorded after weaning (Table S1).

### Vector administration

Each vector was diluted in PBS and aliquoted into single tubes by an independent researcher. To each treatment group was randomly assigned a number 1–8. Each week, 7 KOs and 1 WT pups were injected with one aliquot of each treatment following genotyping at birth, for a total of n = 9 mice per each treatment cohort. Post-natal day 1 (P1) pups were injected with the viral vector or PBS via the superficial temporal vein using a 33-gauge needle (Hamilton). The operator was blinded to both the genotype of the animal and the treatment at the moment of injection. Each vector was administered either at a high dose (HD) of 2.4×10^12^ viral vector genomes (vg) or a low dose (LD) of 3.3×10^11^ vg. KO control and WT control mice were injected with 40 μl saline. Recovered pups were returned to the dam cage.

### Behavioural testing

8-week-old mice underwent rotarod testing and open field testing. Mice were initially trained on the day before performing the tests. On testing day, mice were introduced into the behavioural suite 15 minutes prior starting the tests. The operator remained blinded to the treatment group of the animals during the assessments. The rotarod (Biochrom) was set at a starting speed of 4 rpm with 20 rpm/min acceleration. Time at fall was reported for three consecutive rounds of testing and the best score was used. Mice falling within the first 5 seconds were reported as failing the test. Open field testing was conducted in a plexiglas chamber (27 cm × 27 cm × 27 cm). The mice were allowed to explore the box for 5 minutes before the test. Mice were filmed from the top of the chamber for additional 5 minutes. The analysis of distance and mean speed was carried out using ANY-maze Behavioural Tracking Software v. 4.99 (Stoelting).

### Sample collection

Blood samples were taken from the tail vein at the age of 4 weeks and 8 weeks. The samples were placed into K3-EDTA micro tubes (Sarstedt) and centrifuged for 5 minutes to separate the plasma. Plasma was transferred to a fresh tube, snap frozen and stored at −80°C for future use. Mice were euthanized by terminal transcardial perfusion with PBS. Harvested organs were divided into two halves. One part was fixed in 4% paraformaldehyde (PFA) solution for 48 h at 4°C and moved to a cryoprotective 30% sucrose solution at 4°C. The other half was snap frozen and stored at −80°C for future use. Bone marrow was collected from the femur, flushed with PBS and snap frozen^*^.

^*^Bone marrow sample material from the LD CBA.hGBA cohort was not sufficient to perform the VCN assay. Data not included.

### Histological and immunohistochemical analyses

Fixed organs were cut at 40 μm in thickness at constant temperature of −20°C with a Cryostat Leica CM3050 (Leica Biosystems). CD68, GFAP and LAMP1 markers were used as indexes to evaluate microglia activation, astrogliosis and lysosome accumulation respectively. Immunohistochemical staining was performed as previously described [27]. Briefly, endogenous peroxidase activity was blocked with 1% peroxidase solution in Tris-buffered saline (TBS) for 30 minutes. Non-specific binding sites were blocked with 15% goat serum (Sigma-Aldrich) solution in 0.3% Triton X-100 TBS (TBS-T) for 30 minutes. Sections were incubated with the primary antibody for GCase (1:1000, G4171 Sigma-Aldrich), CD68 (1:2000, ab290 Abcam), GFAP (1:2000, MAB3402 Millipore), LAMP-1^*^ (1:2000, ab24170 Abcam) in 10% goat serum in TBS-T at 4°C overnight. Sections were incubated with secondary biotinilated antibody anti-mouse (BA-9200 Vecotr Lb), anti-rabbit (BA-1000 Vector Lb) or anti-rat (BA-9400 Vector Lb) IgG (1:1000 in TBS-T) for 2 h at room temperature. Sections were incubated with 1:1000 avidine-biotine reagent (Vectastain Elite ABC, Vector Lb) in TBS for 2 h. Immunostaining was developed incubating the sections with 0.05% 3,3′-diaminobenzidine tetrahydrochloride (Sigma-Aldrich). Sections were mounted on chrome-gelatine coated slides, dehydrated in 100% ethanol, cleared in Histo-clear (National Diagnostic) and coverslipped with DPX mountant medium (Thermo Fisher Scientific).

^*^Due to lack of available tissue, LAMP1 staining was performed and quantified in 4 animals/cohort.

Representative sections of visceral organs were mounted onto chrome-gelatine coated slides and dried overnight. Sections were stained with 0.1% Mayer Hematoxylin for 10 minutes and 0.5% Eosin solutions (Sigma-Aldrich). Sections were rinsed in distilled water and dehydrated for 30 seconds in rising concentrations of ethanol, cleared in Histo-clear and coverslipped with DPX mountant medium.

Brain sections were mounted onto chrome-gelatine coated slides and dried overnight. Sections were stained with 0.05% Cresyl Violet solution (VWR) for 30 minutes at 60°C. Slides were rinsed in distilled water and dehydrated in increasing concentrations of ethanol. Sections were cleared in Histo-clear and coverslipped with DPX mountant medium.

Representative images were captured using a Nikon DS-Fi1 camera attached to a Nikon Eclipse E600 microscope.

### Quantitative analysis of staining

To analyze the degree of immunoreactivity for CD68, GFAP, LAMP-1, a semiautomated thresholding image analysis was used with Image-Pro Premier software (Media Cybernetics, MD) [41]. Briefly, slide-scanned images at 10x magnification for stained sections were collected for all animals using a Zeiss Axioscan Z1 slide scanner (Zeiss, Cambridge, United Kingdom). These images were then analysed using Image pro-premier software (Media Cybernetics) for demarcation of anatomical regions of interest and applying appropriate thresholds that selected the foreground immunoreactivity above background. Separate thresholds were used for each antigen and each anatomical region analysed.. The intensity of staining was expressed as percentage of immunoreactivity. Data were reported as average percentage values for each distinct region.

### Stereological analysis

Only age-matched 8-week-old mice were analysed, to ensure a consistent and uniform comparison throughout the stereological investigation. Nissl-stained brain sections were used to conduct stereological analysis with the Stereo Investigator software (MBF Bioscience). Neuron counting was performed using the optical fractionator probe with 150 × 150 μm grid size and 50 × 50 μm counting frame settings. Gundersen coefficient of error was estimated between 0.05 and 0.1.

The average thickness measurement of the S1BF cortical region was calculated using the Cavalieri vertical estimator. 10 parallel lines were traced from the layer 1 of the S1BF region to the corpus callosum and their average length was measured.

### Enzymatic activity assay

Standard GCase enzymatic activity assay was outsourced to XenoGesis (Nottingham, UK) and performed as previously described [26, 27]. Frozen samples were homogenated in distilled water. 30 μl samples were incubated with the synthetic substrate 4-methylumbelliferyl β-D-glucopyranoside (Sigma-Alrich) in 0.15 M citrate/phosphate buffer, pH 5.9 at 37°C for 1 h. The reaction was stopped by adding 200 μl 1 M glycine buffer pH 11. Fluorescence levels of the samples and 4-methylumbelliferone standards were measured. The measurements were conducted in triplicate. GCase enzymatic activity was expressed as nmol/hr/μg.

GCase activity was measured in HEK-293 cell lysate and supernatant using a similar protocol. 48 h post-transfections the cells were harvested. Cell lysate was transferred to a fresh tube. Total protein concentration was determined using the Pierce BCA Protein Assay kit (ThermoFisher) according to the manufacturer’s instructions. GCase enzymatic activity assay was performed as described above.

### Mass spectrometry

Measurement of GlcCer concentration was carried out by XenoGesis Ltd as previously described [26]. Protein concentration of tissue homogenates was quantified using the Pierce BCA Protein Assay kit (ThermoFisher). LC–MS/MS was used to quantify the concentration of GlcCer substrate in each tissue^*^, including each of the different fatty acid forms of the substrate (C:16, C:18, C:20, C:22, C:23, C:24). The substrates were extracted in 10 volumes of methanol containing 25 ng ml − 1 of 15-Hydroxyicosatetraenoic acid (Sigma Aldrich). The samples were shaken for 5 min at room temperature on a Bioshake at 2000 rpm and transferred to the −20°C freezer for a minimum of 2 h. After a 20 min centrifugation at 2500 g, the supernatants were transferred to a 96-well plate and 1 μl injected into the ultra—high performance liquid chromatography–tandem mass spectrometry system. Glycosphingolipid reference standards (Matreya) were also analyzed to confirm analyte identity. The samples were injected onto a Thermo Vanquish UHPLC system operated in partial loop mode and separated on a Phenomenex Luna Omega Polar C18 column (100 Å, 1.6 μm, 2.1 mm × 50 mm) under the following gradient conditions: Initial 80% A, 0.00–0.70 → 0% A; 0.70–1.30 → 0% A; 1.30–1.40 → 80% A; 1.4–1.70 → 80% A, where mobile phase A was Milli-Q H2O with 0.1% FA; phase B was isopropanol/acetonitrile (1:1 v/v) with 0.1% FA and the flow rate was 0.8 mL min − 1. Column and sample temperatures were kept at 65°C and 6°C, respectively. Wash solvent was methanol/acetonitrile/isopropanol/H_2_O (2:1:1:1 v/v). The eluting analytes were detected on a Thermo TSQ Quantiva triple quadrupole mass spectrometer that was equipped with the electrospray ion source and operated in multiple reaction monitoring and negative ion mode with the tune page parameters set to achieve the maximum sensitivity for glycosphingolipids. The data were processed with Xcalibur v4.1. Results were presented as GluCer concentration (μg/g of total protein).

^*^Heart sample material from the LD CAG.hGBA cohort was not sufficient to perform the assay. Data not included.

### Vector copy number analysis

DNA from homogenate samples was isolated using the DNaesy Blood & Tissue kit (QIAGEN). Vector copy number (VCN) was estimated via quantitative PCR using the StepOne Plus Real-Time PCR (Applied Biosystem). The reaction was performed in a final volume of 10 μl with 5 μl iTaq Universal SYBR Green Supermix (Bio-Rad), 100 nM primers (WPRE_F: 5′-tggtgtgcactgtgtttgctga-3′; WPRE_R: 5′-aacataggcgagcagccaaggaaa-3′. Titin_F: 5′-aggatgcctcctgcttaga-3′; Titin_R: 5′-aaacgagcagtgactgag-3′) and 2 μl DNA. Serial dilutions (from 10^8^ to 10 copies/μl) of plasmids containing the WPRE sequence and the murine titin gene were used to construct the standard curves. Each sample and standard was run in duplicate in 96-well plates. The PCR protocol included an initial denaturation step at 95°C for 10 seconds, followed by 40 cycles of denaturation at 95°C for 15 seconds, annealing at 71°C for 1 minute and a melt curve stage. The results were expressed as vector copy number per diploid genome equivalent (VCN/dge).

### Sample size, randomization, and statistics

Samples size analysis and randomization were conducted by an independent statistician consultant. The power of the study was calculated, with estimated minimal sample size n = 8 per group. 9 mice/treatment group were injected to correct for possible complications during injection procedure, sample collection or preparation. To each treatment group was assigned a randomised number 1-to-8 at the moment of sample preparation. The order in which the treatments were administered was randomised, with the order randomly changing each week to minimise bias (Table S2).

Statistical analysis was performed with GraphPad Prism 9.0.1. Data are presented as single points with mean value. All statistical tests and *P* values are reported in Tables S3-S11.

## Supplementary Material

Supplementary_figures_merged_ddae081

Supplementary_legends_ddae081

## References

[1] Pastores GM, Hughes DA. In: Adam M.P., Ardinger H.H., Pagon R.A.. et al. (eds.), GeneReviews((R)). Seattle (WA), 1993.

[2] Nalysnyk L, Rotella P, Simeone JC. et al. Gaucher disease epidemiology and natural history: a comprehensive review of the literature. Hematology 2017;22:65–73. 27762169 10.1080/10245332.2016.1240391

[3] Roh J, Subramanian S, Weinreb NJ. et al. Gaucher disease - more than just a rare lipid storage disease. J Mol Med (Berl) 2022;100:499–518. 35066608 10.1007/s00109-021-02174-z

[4] Dekker N, van Dussen L, Hollak CE. et al. Elevated plasma glucosylsphingosine in Gaucher disease: relation to phenotype, storage cell markers, and therapeutic response. Blood 2011;118:e118–e127. 21868580 10.1182/blood-2011-05-352971 PMC3685900

[5] Mistry PK, Lopez G, Schiffmann R. et al. Gaucher disease: progress and ongoing challenges. Mol Genet Metab 2017;120:8–21. 27916601 10.1016/j.ymgme.2016.11.006 PMC5425955

[6] Roshan, Lal T, Sidransky E. The Spectrum of neurological manifestations associated with Gaucher disease. Diseases 2017;5:10. 10.3390/diseases5010010 PMC5456331 28933363

[7] Charrow J, Andersson HC, Kaplan P. et al. The Gaucher registry: demographics and disease characteristics of 1698 patients with Gaucher disease. Arch Intern Med 2000;160:2835–2843. 11025794 10.1001/archinte.160.18.2835

[8] Goker-Alpan O, Schiffmann R, Park JK. et al. Phenotypic continuum in neuronopathic Gaucher disease: an intermediate phenotype between type 2 and type 3. J Pediatr 2003;143:273–276. 12970647 10.1067/S0022-3476(03)00302-0

[9] Schiffmann R, Sevigny J, Rolfs A. et al. The definition of neuronopathic Gaucher disease. J Inherit Metab Dis 2020;43:1056–1059. 32242941 10.1002/jimd.12235 PMC7540563

[10] Daykin EC, Ryan E, Sidransky E. Diagnosing neuronopathic Gaucher disease: new considerations and challenges in assigning Gaucher phenotypes. Mol Genet Metab 2021;132:49–58. 33483255 10.1016/j.ymgme.2021.01.002 PMC7884077

[11] Wong K, Sidransky E, Verma A. et al. Neuropathology provides clues to the pathophysiology of Gaucher disease. Mol Genet Metab 2004;82:192–207. 15234332 10.1016/j.ymgme.2004.04.011

[12] Horowitz M, Braunstein H, Zimran A. et al. Lysosomal functions and dysfunctions: molecular and cellular mechanisms underlying Gaucher disease and its association with Parkinson disease. Adv Drug Deliv Rev 2022;187:114402. 35764179 10.1016/j.addr.2022.114402

[13] Sidransky E, Lopez G. The link between the GBA gene and parkinsonism. Lancet Neurol 2012;11:986–998. 23079555 10.1016/S1474-4422(12)70190-4 PMC4141416

[14] Revel-Vilk S, Szer J, Mehta A. et al. How we manage Gaucher disease in the era of choices. Br J Haematol 2018;182:467–480. 29808905 10.1111/bjh.15402

[15] Zimran A, Elstein D. Management of Gaucher disease: enzyme replacement therapy. Pediatr Endocrinol Rev 2014;12:82–87. 25345089

[16] Sam R, Ryan E, Daykin E. et al. Current and emerging pharmacotherapy for Gaucher disease in pediatric populations. Expert Opin Pharmacother 2021;22:1489–1503. 33711910 10.1080/14656566.2021.1902989 PMC8373623

[17] Hudry E, Vandenberghe LH. Therapeutic AAV gene transfer to the nervous system: a clinical reality. Neuron 2019;102:263. 30946822 10.1016/j.neuron.2019.03.020

[18] Deverman BE, Ravina BM, Bankiewicz KS. et al. Gene therapy for neurological disorders: progress and prospects. Nat Rev Drug Discov 2018;17:767. 30206384 10.1038/nrd.2018.158

[19] Gray SJ, Matagne V, Bachaboina L. et al. Preclinical differences of intravascular AAV9 delivery to neurons and glia: a comparative study of adult mice and nonhuman primates. Mol Ther 2011;19:1058–1069. 21487395 10.1038/mt.2011.72 PMC3129805

[20] Hudry E, Andres-Mateos E, Lerner EP. et al. Efficient gene transfer to the central nervous system by single-stranded Anc80L65. Mol Ther Methods Clin Dev 2018;10:197–209. 30109242 10.1016/j.omtm.2018.07.006 PMC6083902

[21] Mattar CN, Wong AM, Hoefer K. et al. Systemic gene delivery following intravenous administration of AAV9 to fetal and neonatal mice and late-gestation nonhuman primates. FASEB J 2015;29:3876–3888. 26062602 10.1096/fj.14-269092 PMC4560173

[22] Rahim AA, Wong AM, Hoefer K. et al. Intravenous administration of AAV2/9 to the fetal and neonatal mouse leads to differential targeting of CNS cell types and extensive transduction of the nervous system. FASEB J 2011;25:3505–3518. 21746868 10.1096/fj.11-182311

[23] Wang DB, Dayton RD, Henning PP. et al. Expansive gene transfer in the rat CNS rapidly produces amyotrophic lateral sclerosis relevant sequelae when TDP-43 is overexpressed. Mol Ther 2010;18:2064–2074. 20877346 10.1038/mt.2010.191 PMC2997590

[24] Mendell JR, Al-Zaidy S, Shell R. et al. Single-dose gene-replacement therapy for spinal muscular atrophy *N*. Engl J Med 2017;377:1713–1722. 10.1056/NEJMoa1706198 29091557

[25] Mendell JR, Al-Zaidy SA, Lehman KJ. et al. Five-year extension results of the phase 1 START trial of Onasemnogene Abeparvovec in spinal muscular atrophy. JAMA Neurol 2021;78:834–841. 33999158 10.1001/jamaneurol.2021.1272 PMC8129901

[26] Massaro G, Mattar CNZ, Wong AMS. et al. Fetal gene therapy for neurodegenerative disease of infants. Nat Med 2018;24:1317–1323. 30013199 10.1038/s41591-018-0106-7 PMC6130799

[27] Massaro G, Hughes MP, Whaler SM. et al. Systemic AAV9 gene therapy using the synapsin I promoter rescues a mouse model of neuronopathic Gaucher disease but with limited cross-correction potential to astrocytes. Hum Mol Genet 2020;29:1933–1949. 31919491 10.1093/hmg/ddz317 PMC7390934

[28] Enquist IB, Lo Bianco C, Ooka A. et al. Murine models of acute neuronopathic Gaucher disease. Proc Natl Acad Sci USA 2007;104:17483–17488. 17954912 10.1073/pnas.0708086104 PMC2077282

[29] Ertl HCJ . Immunogenicity and toxicity of AAV gene therapy. Front Immunol 2022;13:975803. 36032092 10.3389/fimmu.2022.975803 PMC9411526

[30] Gray SJ, Nagabhushan Kalburgi S, McCown TJ. et al. Global CNS gene delivery and evasion of anti-AAV-neutralizing antibodies by intrathecal AAV administration in non-human primates. Gene Ther 2013;20:450–459. 23303281 10.1038/gt.2012.101 PMC3618620

[31] Hinderer C, Bell P, Vite CH. et al. Widespread gene transfer in the central nervous system of cynomolgus macaques following delivery of AAV9 into the cisterna magna. Mol Ther Methods Clin Dev 2014;1:14051. 26052519 10.1038/mtm.2014.51 PMC4448732

[32] Ohno K, Samaranch L, Hadaczek P. et al. Kinetics and MR-based monitoring of AAV9 vector delivery into cerebrospinal fluid of nonhuman primates. Mol Ther Methods Clin Dev 2019;13:47–54. 30666308 10.1016/j.omtm.2018.12.001 PMC6330508

[33] Samaranch L, Bringas J, Pivirotto P. et al. Cerebellomedullary cistern delivery for AAV-based gene therapy: a technical note for nonhuman primates. Hum Gene Ther Methods 2016;27:13–16. 26757202 10.1089/hgtb.2015.129 PMC4761826

[34] Kishimoto TK, Samulski RJ. Addressing high dose AAV toxicity - 'one and done' or 'slower and lower'? Expert Opin Biol Ther 2022;22:1067–1071. 35373689 10.1080/14712598.2022.2060737

[35] Guillou J, de Pellegars A, Porcheret F. et al. Fatal thrombotic microangiopathy case following adeno-associated viral SMN gene therapy. Blood Adv 2022;6:4266–4270. 35584395 10.1182/bloodadvances.2021006419 PMC9327533

[36] McEachern KA, Nietupski JB, Chuang WL. et al. AAV8-mediated expression of glucocerebrosidase ameliorates the storage pathology in the visceral organs of a mouse model of Gaucher disease. J Gene Med 2006;8:719–729. 16528760 10.1002/jgm.901

[37] Du S, Ou H, Cui R. et al. Delivery of Glucosylceramidase Beta gene using AAV9 vector therapy as a treatment strategy in mouse models of Gaucher disease. Hum Gene Ther 2019;30:155–167. 30122074 10.1089/hum.2018.072

[38] Baruteau J, Waddington SN. Fetal gene therapy for neurodegenerative lysosomal storage diseases. J Inherit Metab Dis 2019;42:391–393. 30715735 10.1002/jimd.12018

[39] Waddington SN, Peranteau WH, Rahim AA. et al. Fetal gene therapy. J Inherit Metab Dis 2024;47:192–210. 37470194 10.1002/jimd.12659 PMC10799196

[40] Cohen JL, Chakraborty P, Fung-Kee-Fung K. et al. In utero enzyme-replacement therapy for infantile-onset Pompe's disease. N Engl J Med 2022;135:S33. 10.1056/NEJMoa2200587 PMC10794051 36351280

[41] Nelvagal HR, Dearborn JT, Ostergaard JR. et al. Spinal manifestations of CLN1 disease start during the early postnatal period. Neuropathol Appl Neurobiol 2021;47:251–267. 32841420 10.1111/nan.12658 PMC7867600

