# Supplementary material for: Comparison of different promoters to improve AAV vector-mediated gene therapy for neuronopathic Gaucher disease
Source: Hum Mol Genet. 2024 May 16;33(17):1467–80. doi: 10.1093/hmg/ddae081 (PMC11336133; doi:10.1093/hmg/ddae081)
Supplement: Supplementary_figures_merged_ddae081 [file supplementary_figures_merged_ddae081.pdf]

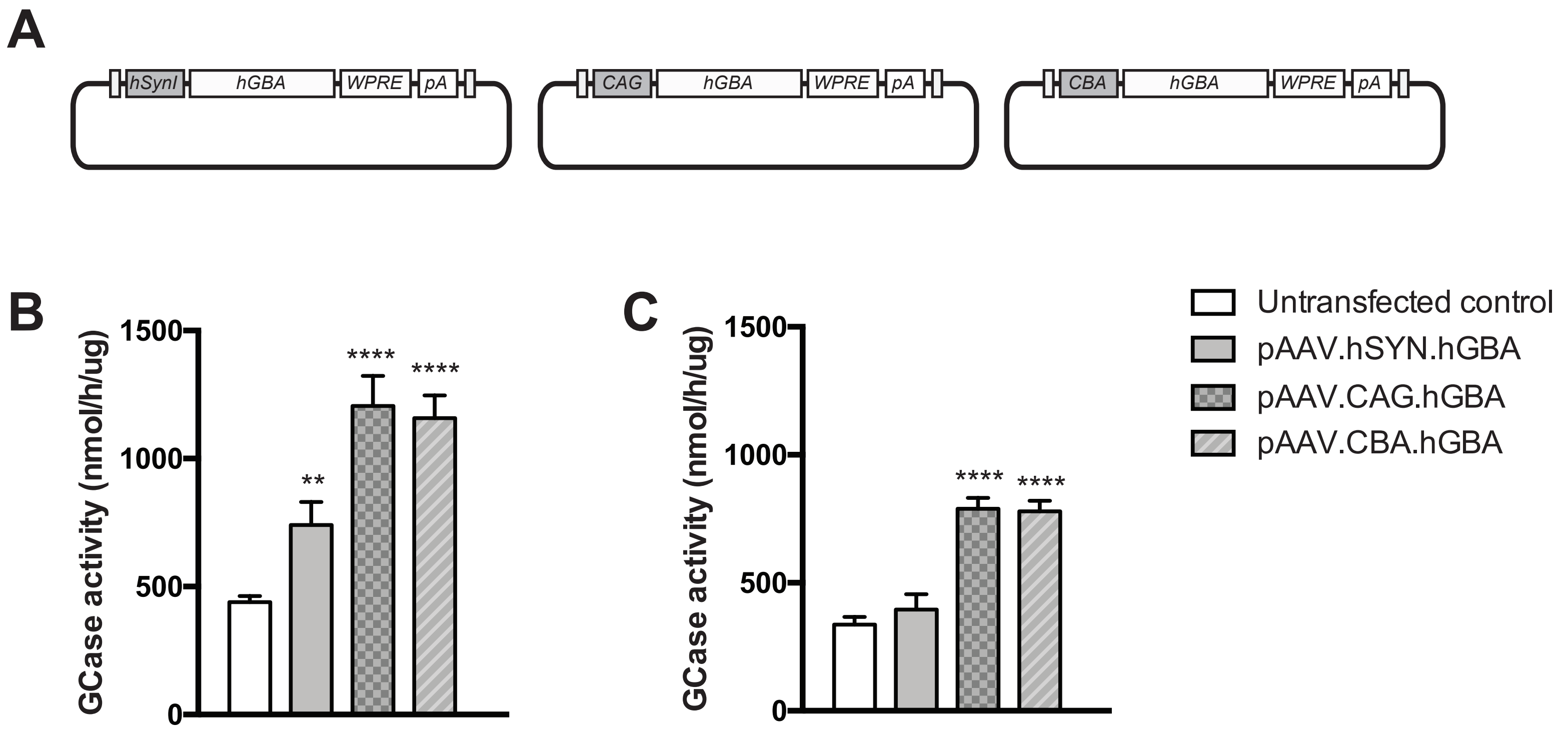
A
hSynI
hGBA
WPRE
pA
CAG
hGBA
WPRE
pA
CBA
hGBA
WPRE
pA
B
GCase activity (nmol/h/ug)
1500
1000
500
0
**
****
****
C
GCase activity (nmol/h/ug)
1500
1000
500
0
****
****
Untransfected control
pAAV.hSYN.hGBA
pAAV.CAG.hGBA
pAAV.CBA.hGBA

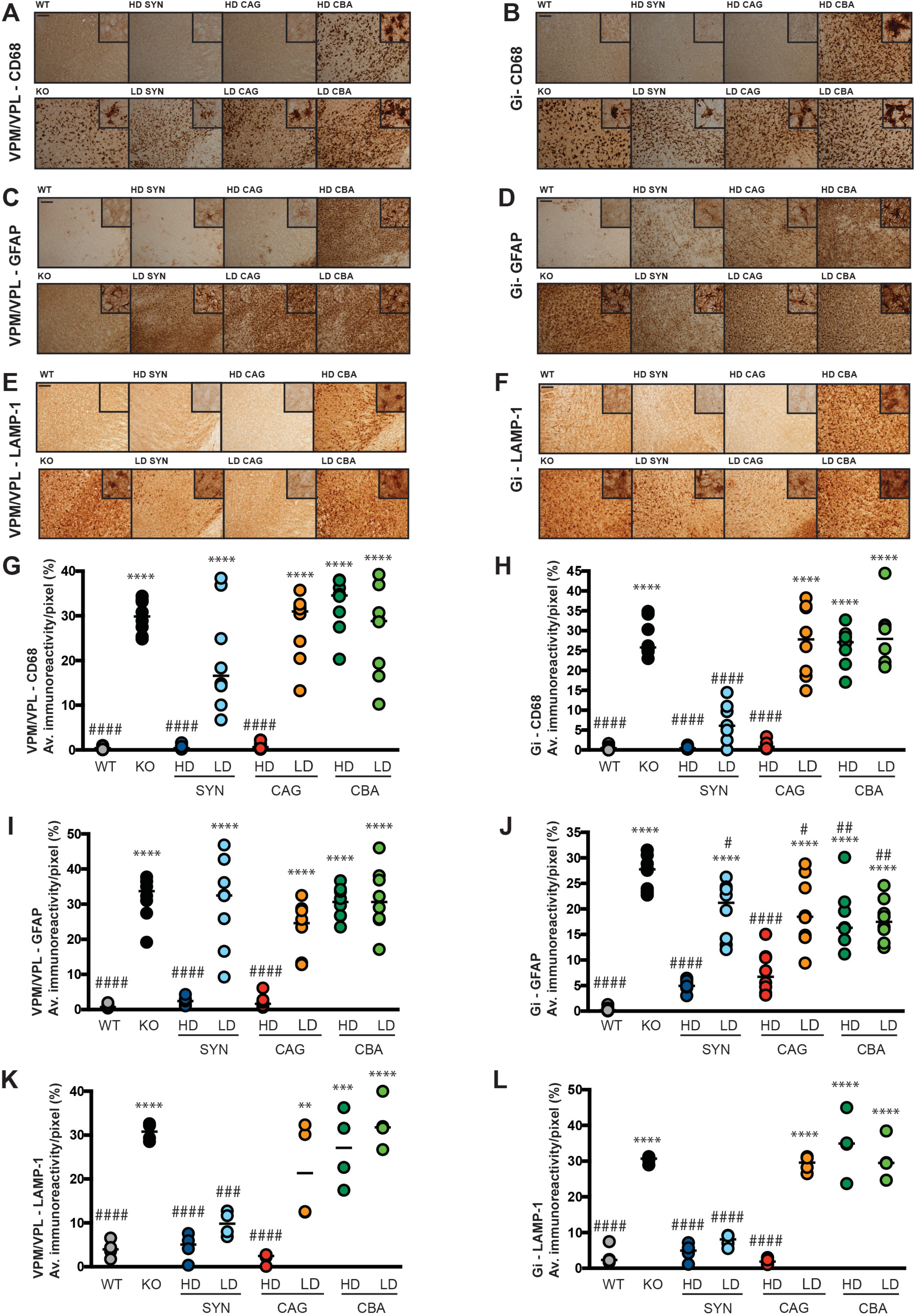

A
VPM/VPL - CD68
WT
HD SYN
HD CAG
HD CBA
KO
LD SYN
LD CAG
LD CBA
B
Gi- CD68
C
VPM/VPL - GFAP
D
Gi- GFAP
E
VPM/VPL - LAMP-1
F
Gi - LAMP-1
G
VPM/VPL - CD68
Av. immunoreactivity/pixel (%)
H
Gi - CD68
Av. immunoreactivity/pixel (%)
I
VPM/VPL - GFAP
Av. immunoreactivity/pixel (%)
J
Gi - GFAP
Av. immunoreactivity/pixel (%)
K
VPM/VPL - LAMP-1
Av. immunoreactivity/pixel (%)
L
Gi - LAMP-1
Av. immunoreactivity/pixel (%)
WT KO HD LD HD LD HD LD
SYN CAG CBA

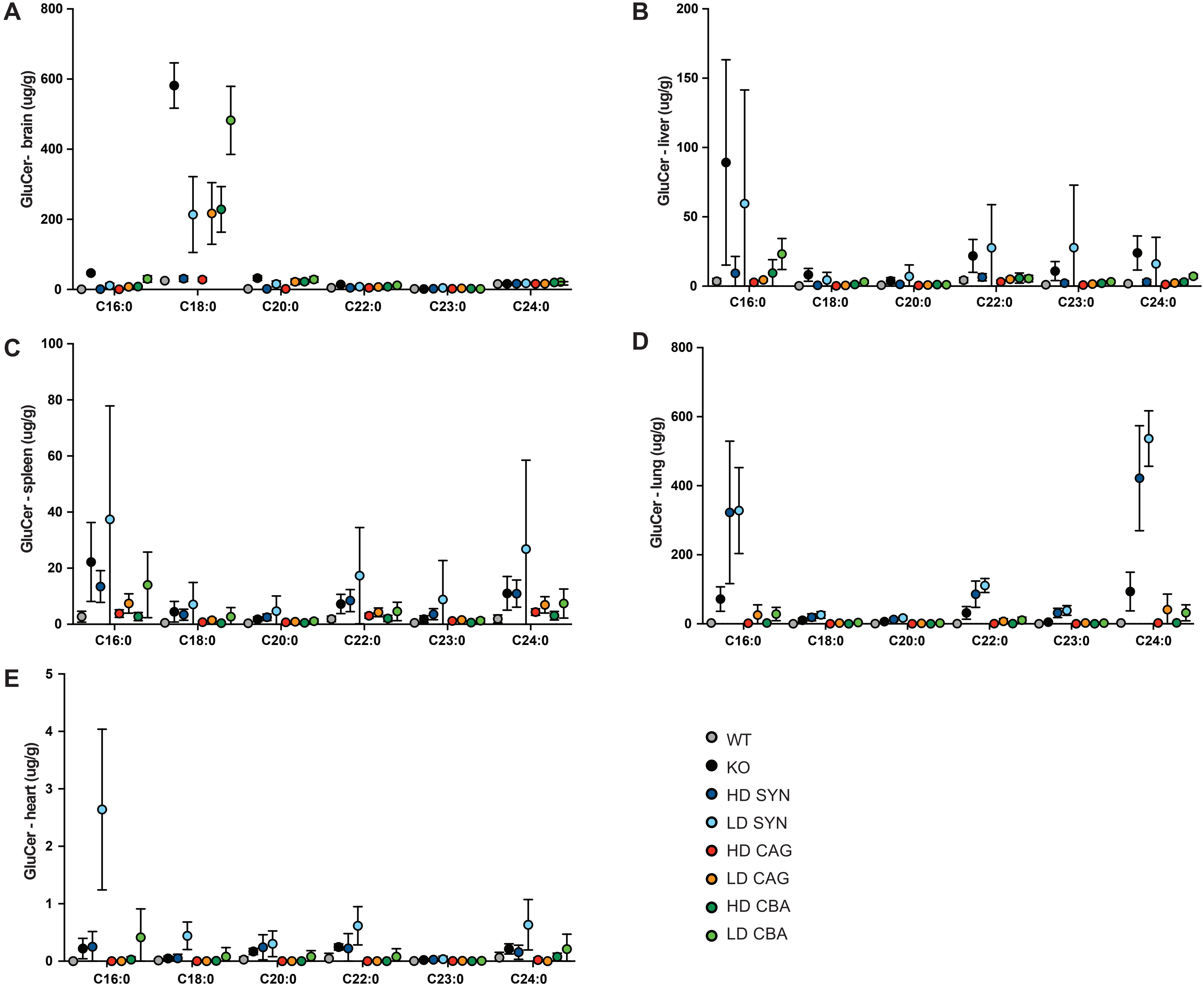

A
GluCer- brain (ug/g)
B
GluCer - liver (ug/g)
C
GluCer - spleen (ug/g)
D
GluCer - lung (ug/g)
E
GluCer - heart (ug/g)
C16:0
C18:0
C20:0
C22:0
C23:0
C24:0
WT
KO
HD SYN
LD SYN
HD CAG
LD CAG
HD CBA
LD CBA

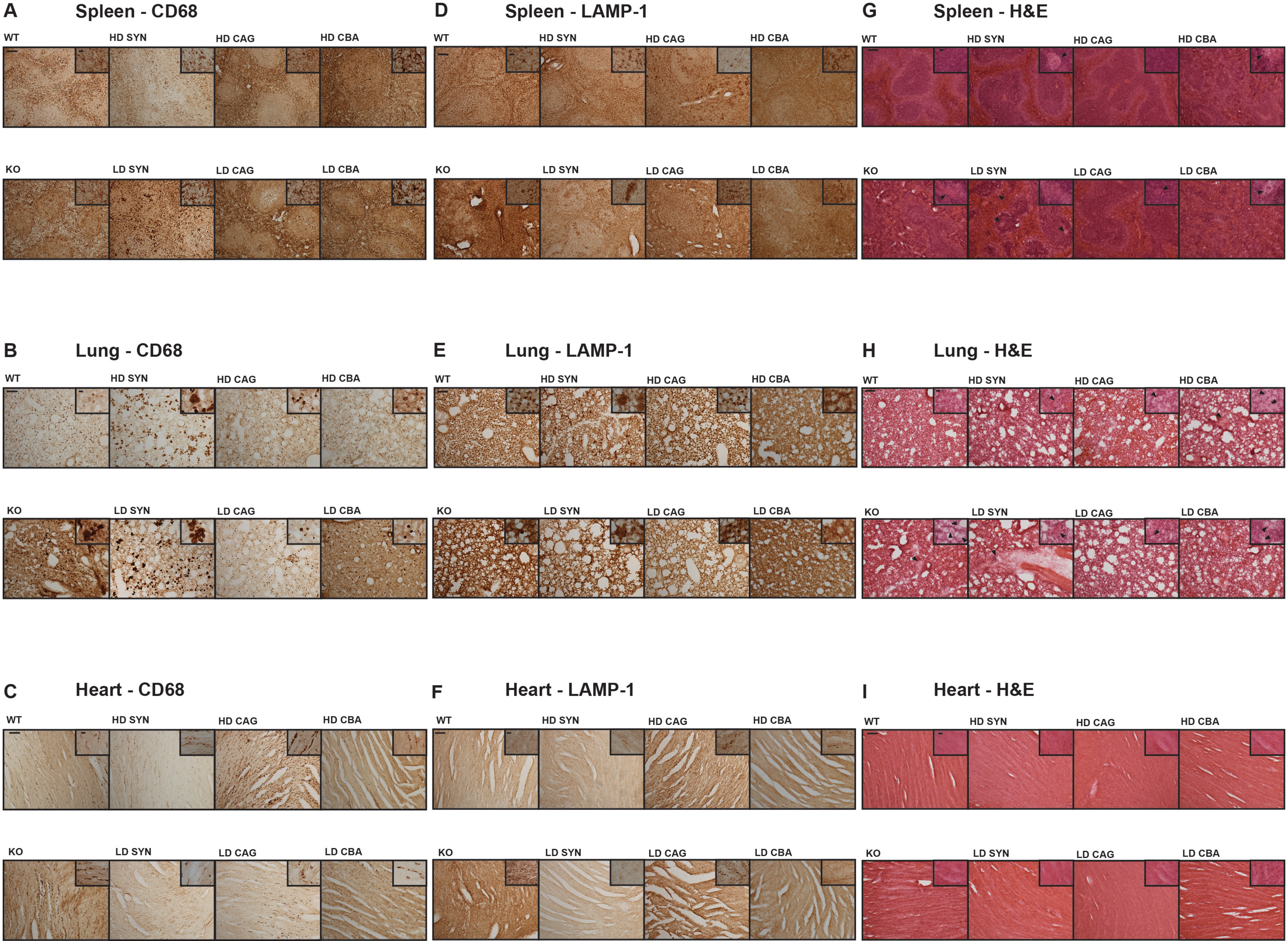
A
Spleen - CD68
D
Spleen - LAMP-1
G
Spleen - H&E
B
Lung - CD68
E
Lung - LAMP-1
H
Lung - H&E
C
Heart - CD68
F
Heart - LAMP-1
I
Heart - H&E
WT
HD SYN
HD CAG
HD CBA
KO
LD SYN
LD CAG
LD CBA

Week	Cohort treatment	Sex
1	Trt 1	M
1	Trt 2	M
1	Trt 3	M
1	Trt 4	F
1	Trt 5	Undetermined
1	Trt 6	M
1	Trt 7	Undetermined
1	Trt 8	F
2	Trt 1	M
2	Trt 2	F
2	Trt 3	F
2	Trt 4	F
2	Trt 5	Undetermined
2	Trt 6	F
2	Trt 7	F
2	Trt 8	F
3	Trt 1	M
3	Trt 2	M
3	Trt 3	F
3	Trt 4	M
3	Trt 5	F
3	Trt 6	F
3	Trt 7	M
3	Trt 8	M
4	Trt 1	M
4	Trt 2	M
4	Trt 3	F
4	Trt 4	M
4	Trt 5	Undetermined
4	Trt 6	M
4	Trt 7	Undetermined
4	Trt 8	M
5	Trt 1	M
5	Trt 2	F
5	Trt 3	M
5	Trt 4	M
5	Trt 5	M
5	Trt 6	F
5	Trt 7	Undetermined
5	Trt 8	M

Week	Cohort treatment	Sex
6	Trt 1	M
6	Trt 2	M
6	Trt 3	M
6	Trt 4	M
6	Trt 5	F
6	Trt 6	F
6	Trt 7	Undetermined
6	Trt 8	M
7	Trt 1	F
7	Trt 2	F
7	Trt 3	M
7	Trt 4	F
7	Trt 5	Undetermined
7	Trt 6	F
7	Trt 7	M
7	Trt 8	M
8	Trt 1	F
8	Trt 2	M
8	Trt 3	M
8	Trt 4	F
8	Trt 5	Undetermined
8	Trt 6	M
8	Trt 7	
8	Trt 8	M
9	Trt 1	F
9	Trt 2	M
9	Trt 3	M
9	Trt 4	M
9	Trt 5	Undetermined
9	Trt 6	F
9	Trt 7	Undetermined
9	Trt 8	F

Week	Cohort treatment	Tube
1	Trt 4	41
1	Trt 6	6.1
1	Trt 1	1.1
1	Trt 5	5.1
1	Trt 7	7.1
1	Trt 3	3.1
1	Trt 2	2.1
2	Trt 1	1.2
2	Trt 3	3.2
2	Trt 5	5.2
2	Trt 2	2.2
2	Trt 4	4.2
2	Trt 7	7.2
2	Trt 6	6.2
3	Trt 6	6.3
3	Trt 1	1.3
3	Trt 3	3.3
3	Trt 7	7.3
3	Trt 2	2.3
3	Trt 5	5.3
3	Trt 4	4.3
4	Trt 3	3.4
4	Trt 5	5.4
4	Trt 7	7.4
4	Trt 4	4.4
4	Trt 6	6.4
4	Trt 2	2.4
4	Trt 1	1.4
5	Trt 2	2.5
5	Trt 4	4.5
5	Trt 6	6.5
5	Trt 3	3.5
5	Trt 5	5.5
5	Trt 1	1.5
5	Trt 7	7.5

Week	Cohort treatment	Tube
6	Trt 5	5.6
6	Trt 7	7.6
6	Trt 2	2.6
6	Trt 6	6.6
6	Trt 1	1.6
6	Trt 4	4.6
6	Trt 3	3.6
7	Trt 7	7.7
7	Trt 2	2.7
7	Trt 4	4.7
7	Trt 1	1.7
7	Trt 3	3.7
7	Trt 6	6.7
7	Trt 5	5.7
8	Trt 2	2.8
8	Trt 1	1.8
8	Trt 3	3.8
8	Trt 4	4.8
8	Trt 6	6.8
8	Trt 5	5.8
8	Trt 7	7.8
9	Trt 1	1.9
9	Trt 2	2.9
9	Trt 3	3.9
9	Trt 4	4.9
9	Trt 5	5.9
9	Trt 6	6.9
9	Trt 7	7.9

	Treatment assignment
Trt 1	HD SYN
Trt 2	LD SYN
Trt 3	HD CAG
Trt 4	HD CBA
Trt 5	LD CBA
Trt 6	LD CAG
Trt 7	Untreated KO - PBS
Trt 8	WT - PBS

Figure 1

Survival curves

Long-rank (Mantel-Cox)

	P value	Summary
KO vs. WT	<0.0001	****
KO vs. HD SYN	<0.0001	****
KO vs. LD SYN	<0.0001	****
KO vs. HD CAG	<0.0001	****
KO vs. LD CAG	<0.0001	****
KO vs. HD CBA	<0.0001	****
KO vs. LD CBA	0.0101	*

Weekly body weights

One-way ANOVA, Tukey's multiple comparison test

	Week 1		Week 2		Week 3		Week 4		Week 5		Week 6		Week 7		Week 8	
	P value	Summary	P value	Summary	P value	Summary	P value	Summary	P value	Summary	P value	Summary	P value	Summary	P value	Summary
HD SYN vs. LD SYN	0.8797	ns	0.9089	ns	0.7234	ns	0.9638	ns	0.9638	ns	0.9982	ns	0.987	ns	0.9861	ns
HD SYN vs. HD CAG	0.9846	ns	> 0.9999	ns	0.9997	ns	0.9998	ns	0.9998	ns	0.9981	ns	> 0.9999	ns	> 0.9999	ns
HD SYN vs. HD CBA	0.8973	ns	> 0.9999	ns	0.8477	ns	0.7408	ns	0.7408	ns						
HD SYN vs. LD CBA	0.8448	ns	0.998	ns												
HD SYN vs. LD CAG	0.1673	ns	0.9197	ns	0.9263	ns	0.9862	ns	0.9862	ns	0.9341	ns	0.8891	ns	0.8739	ns
HD SYN vs. KO	0.9933	ns	0.8977	ns												
HD SYN vs. WT	0.6208	ns	0.2893	ns	0.0698	ns	0.0908	ns	0.0908	ns	0.248	ns	0.339	ns	0.3867	ns
LD SYN vs. HD CAG	0.9998	ns	0.9537	ns	0.5039	ns	0.8749	ns	0.8749	ns	>0.9999	ns	0.9658	ns	0.9943	ns
LD SYN vs. HD CBA	> 0.9999	ns	0.975	ns	0.1037	ns	0.3393	ns	0.3393	ns						
LD SYN vs. LD CBA	> 0.9999	ns	0.5377	ns												
LD SYN vs. LD CAG	0.8863	ns	> 0.9999	ns	0.9974	ns	0.6749	ns	0.6749	ns	0.9846	ns	0.986	ns	0.984	ns
LD SYN vs. KO	0.9994	ns	0.1783	ns												
LD SYN vs. WT	0.9997	ns	0.9519	ns	0.685	ns	0.4192	ns	0.4192	ns	0.1413	ns	0.1382	ns	0.212	ns
HD CAG vs. HD CBA	> 0.9999	ns	> 0.9999	ns	0.944	ns	0.8435	ns	0.8435	ns						
HD CAG vs. LD CBA	0.9992	ns	0.9887	ns												
HD CAG vs. LD CAG	0.6397	ns	0.9606	ns	0.7826	ns	0.9988	ns	0.9988	ns	0.9827	ns	0.834	ns	0.9044	ns
HD CAG vs. KO	> 0.9999	ns	0.792	ns												
HD CAG vs. WT	0.9822	ns	0.3578	ns	0.0263	*	0.0384	*	0.0384	*	0.1234	ns	0.3823	ns	0.2912	ns
HD CBA vs. LD CBA	> 0.9999	ns	0.9766	ns					0.9502	ns						
HD CBA vs. LD CAG	0.867	ns	0.9794	ns	0.2554	ns	0.9502	ns								
HD CBA vs. KO	0.9997	ns	0.7253	ns												
HD CBA vs. WT	0.9995	ns	0.4293	ns	0.002	**	0.01	*	0.01	*						
LD CBA vs. LD CAG	0.939	ns	0.5592	ns												
LD CBA vs. KO	0.9982	ns	0.998	ns												
LD CBA vs. WT	> 0.9999	ns	0.0699	ns												
LD CAG vs. KO	0.6123	ns	0.1905	ns												
LD CAG vs. WT	0.9895	ns	0.9439	ns	0.4018	ns	0.014	*	0.014	*	0.0892	ns	0.1234	ns	0.1772	ns
KO vs. WT	0.9737	ns	0.0112	*												

Δ average body weight vs. WT

Difference in least square means and 95% Cis, unadjusted analysis

	P value	Summary
WT vs. HD SYN	0.0831	ns
WT vs. LD SYN	0.0045	*
WT vs. HD CAG	0.0682	ns
WT vs. LD CAG	0.0085	*

GCase activity 1M

One-way ANOVA, Tukey's multiple comparison test

	P value	Summary
WT vs. HD SYN	0.9807	ns
WT vs. LD SYN	0.9874	ns
WT vs. HD CAG	<0.0001	****
WT vs. LD CAG	0.0161	*
WT vs. HD CBA	0.7157	ns

GCase activity end stage

One-way ANOVA, Tukey's multiple comparison test

	P value	Summary
HD SYN vs. LD SYN	> 0.9999	ns
HD SYN vs. HD CAG	< 0.0001	****
HD SYN vs. LD CAG	0.1043	ns
HD SYN vs. HD CBA	< 0.0001	****
HD SYN vs. LD CBA	0.6502	ns
HD SYN vs. WT	> 0.9999	ns
HD SYN vs. KO	> 0.9999	ns
LD SYN vs. HD CAG	< 0.0001	****
LD SYN vs. LD CAG	0.0774	ns
LD SYN vs. HD CBA	< 0.0001	****
LD SYN vs. LD CBA	0.6173	ns
LD SYN vs. WT	> 0.9999	ns
LD SYN vs. KO	> 0.9999	ns
HD CAG vs. LD CAG	< 0.0001	****
HD CAG vs. HD CBA	0.0711	ns
HD CAG vs. LD CBA	< 0.0001	****
HD CAG vs. WT	< 0.0001	****
HD CAG vs. KO	< 0.0001	****
LD CAG vs. HD CBA	0.0131	*
LD CAG vs. LD CBA	0.9677	ns
LD CAG vs. WT	0.1206	ns
LD CAG vs. KO	0.1204	ns
HD CBA vs. LD CBA	0.0007	***
HD CBA vs. WT	< 0.0001	****
HD CBA vs. KO	< 0.0001	****
LD CBA vs. WT	0.734	ns
LD CBA vs. KO	0.6893	ns
WT vs. KO	> 0.9999	ns

Figure 2

Average immunereactivity/pixel

One-way ANOVA, Tukey's multiple comparison test

	CD68		GFAP		LAMP	
	P value	Summary	P value	Summary	P value	Summary
HD SYN vs. LD SYN	0.0007	***	0.0129	*	> 0.9999	ns
HD SYN vs. HD CAG	> 0.9999	ns	> 0.9999	ns	> 0.9999	ns
HD SYN vs. LD CAG	< 0.0001	****	0.0026	**	0.0008	***
HD SYN vs. HD CBA	< 0.0001	****	0.0043	**	0.0088	**
HD SYN vs. LD CBA	< 0.0001	****	0.0004	***	0.0103	*
HD SYN vs. WT	> 0.9999	ns	> 0.9999	ns	> 0.9999	ns
HD SYN vs. KO	< 0.0001	****	< 0.0001	****	0.0005	***
LD SYN vs. HD CAG	0.0009	***	0.018	*	0.5612	ns
LD SYN vs. LD CAG	> 0.9999	ns	> 0.9999	ns	0.1139	ns
LD SYN vs. HD CBA	> 0.9999	ns	> 0.9999	ns	0.9743	ns
LD SYN vs. LD CBA	> 0.9999	ns	> 0.9999	ns	> 0.9999	ns
LD SYN vs. WT	0.0008	***	0.0131	*	> 0.9999	ns
LD SYN vs. KO	0.5354	ns	0.3874	ns	0.0762	ns
HD CAG vs. LD CAG	< 0.0001	****	0.0037	**	0.0002	***
HD CAG vs. HD CBA	< 0.0001	****	0.006	**	0.0023	**
HD CAG vs. LD CBA	< 0.0001	****	0.0006	***	0.0027	**
HD CAG vs. WT	> 0.9999	ns	> 0.9999	ns	> 0.9999	ns
HD CAG vs. KO	< 0.0001	****	< 0.0001	****	0.0001	***
LD CAG vs. HD CBA	> 0.9999	ns	> 0.9999	ns	> 0.9999	ns
LD CAG vs. LD CBA	> 0.9999	ns	> 0.9999	ns	> 0.9999	ns
LD CAG vs. WT	< 0.0001	****	0.0026	**	0.0013	**
LD CAG vs. KO	> 0.9999	ns	> 0.9999	ns	> 0.9999	ns
HD CBA vs. LD CBA	> 0.9999	ns	> 0.9999	ns	> 0.9999	ns
HD CBA vs. WT	< 0.0001	****	0.0043	**	0.0143	*
HD CBA vs. KO	> 0.9999	ns	0.8954	ns	> 0.9999	ns
LD CBA vs. WT	< 0.0001	****	0.0004	***	0.0168	*
LD CBA vs. KO	> 0.9999	ns	> 0.9999	ns	> 0.9999	ns
WT vs. KO	< 0.0001	****	< 0.0001	****	0.009	***

GCase activity

One-way ANOVA, Tukey's multiple comparison test

	P value	Summary
HD SYN vs. LD SYN	< 0.0001	****
HD SYN vs. HD CAG	< 0.0001	****
HD SYN vs. LD CAG	< 0.0001	****
HD SYN vs. HD CBA	< 0.0001	****
HD SYN vs. LD CBA	< 0.0001	****
HD SYN vs. WT	< 0.0001	****
HD SYN vs. KO	< 0.0001	****
LD SYN vs. HD CAG	0.0136	*
LD SYN vs. LD CAG	0.8114	ns
LD SYN vs. HD CBA	0.9987	ns
LD SYN vs. LD CBA	0.2925	ns
LD SYN vs. WT	0.0084	**
LD SYN vs. KO	0.1847	ns
HD CAG vs. LD CAG	0.0001	***
HD CAG vs. HD CBA	0.0023	**
HD CAG vs. LD CBA	< 0.0001	****
HD CAG vs. WT	> 0.9999	ns
HD CAG vs. KO	< 0.0001	****
LD CAG vs. HD CBA	0.9867	ns
LD CAG vs. LD CBA	0.9894	ns
LD CAG vs. WT	< 0.0001	****
LD CAG vs. KO	0.9566	ns
HD CBA vs. LD CBA	0.6687	ns
HD CBA vs. WT	0.0012	**
HD CBA vs. KO	0.5093	ns
LD CBA vs. WT	< 0.0001	****
LD CBA vs. KO	> 0.9999	ns
WT vs. KO	< 0.0001	****

GluCer

One-way ANOVA, Tukey's multiple comparison test

	P value	Summary
HD SYN vs. LD SYN	< 0.0001	****
HD SYN vs. HD CAG	> 0.9999	ns
HD SYN vs. LD CAG	< 0.0001	****
HD SYN vs. HD CBA	< 0.0001	****
HD SYN vs. LD CBA	< 0.0001	****
HD SYN vs. WT	> 0.9999	ns
HD SYN vs. KO	< 0.0001	****
LD SYN vs. HD CAG	< 0.0001	****
LD SYN vs. LD CAG	> 0.9999	ns
LD SYN vs. HD CBA	0.9998	ns
LD SYN vs. LD CBA	< 0.0001	****
LD SYN vs. WT	< 0.0001	****
LD SYN vs. KO	< 0.0001	****
HD CAG vs. LD CAG	< 0.0001	****
HD CAG vs. HD CBA	< 0.0001	****
HD CAG vs. LD CBA	< 0.0001	****
HD CAG vs. WT	> 0.9999	ns
HD CAG vs. KO	< 0.0001	****
LD CAG vs. HD CBA	> 0.9999	ns
LD CAG vs. LD CBA	< 0.0001	****
LD CAG vs. WT	< 0.0001	****
LD CAG vs. KO	< 0.0001	****
HD CBA vs. LD CBA	< 0.0001	****
HD CBA vs. WT	< 0.0001	****
HD CBA vs. KO	< 0.0001	****
LD CBA vs. WT	< 0.0001	****
LD CBA vs. KO	0.0824	ns
WT vs. KO	< 0.0001	****

Figure 3

Cortical thickness

	One-way ANOVA, Tukey's multiple comparison test	
	P value	Summary
HD SYN vs. HD CAG	0.9715	ns
HD SYN vs. WT	0.997	ns
HD SYN vs. LD SYN	0.0092	**
HD SYN vs. LD CAG	0.0419	*
HD CAG vs. WT	0.8733	ns
HD CAG vs. LD SYN	0.0021	**
HD CAG vs. LD CAG	0.0144	*
WT vs. LD SYN	0.0197	*
WT vs. LD CAG	0.0725	ns
LD SYN vs. LD CAG	> 0.9999	ns

Neuron count

	One-way ANOVA, Tukey's multiple comparison test	
	P value	Summary
HD SYN vs. HD CAG	0.9992	ns
HD SYN vs. WT	0.9206	ns
HD SYN vs. LD SYN	< 0.0001	****
HD SYN vs. LD CAG	< 0.0001	****
HD CAG vs. WT	0.8208	ns
HD CAG vs. LD SYN	< 0.0001	****
HD CAG vs. LD CAG	0.0001	***
WT vs. LD SYN	< 0.0001	****
WT vs. LD CAG	< 0.0001	****
LD SYN vs. LD CAG	0.6659	ns

Rota rod time

	One-way ANOVA, Tukey's multiple comparison test	
	P value	Summary
HD SYN vs. LD SYN	0.9612	ns
HD SYN vs. HD CAG	0.8952	ns
HD SYN vs. LD CAG	0.1568	ns
HD SYN vs. WT	0.7911	ns
LD SYN vs. HD CAG	0.9998	ns
LD SYN vs. LD CAG	0.0607	ns
LD SYN vs. WT	0.9945	ns
HD CAG vs. LD CAG	0.0357	*
HD CAG vs. WT	0.9993	ns
LD CAG vs. WT	0.024	*

Open field distance

	One-way ANOVA, Tukey's multiple comparison test	
	P value	Summary
HD SYN vs. LD SYN	0.4866	ns
HD SYN vs. HD CAG	> 0.9999	ns
HD SYN vs. LD CAG	0.5657	ns
HD SYN vs. WT	0.9998	ns
LD SYN vs. HD CAG	0.4439	ns
LD SYN vs. LD CAG	0.9991	ns
LD SYN vs. WT	0.3638	ns
HD CAG vs. LD CAG	0.5383	ns
HD CAG vs. WT	0.9999	ns
LD CAG vs. WT	0.4724	ns

Open field mean speed

	One-way ANOVA, Tukey's multiple comparison test	
	P value	Summary
HD SYN vs. LD SYN	0.4845	ns
HD SYN vs. HD CAG	> 0.9999	ns
HD SYN vs. LD CAG	0.5662	ns
HD SYN vs. WT	0.9998	ns
LD SYN vs. HD CAG	0.4296	ns
LD SYN vs. LD CAG	0.9991	ns
LD SYN vs. WT	0.3659	ns
HD CAG vs. LD CAG	0.5289	ns
HD CAG vs. WT	> 0.9999	ns
LD CAG vs. WT	0.4763	ns

Figure 4

Adjusted average liver weight

One-way ANOVA, Tukey's multiple comparison test

	P value	Summary
HD SYN vs. LD SYN	> 0.9999	ns
HD SYN vs. HD CAG	> 0.9999	ns
HD SYN vs. LD CAG	0.9986	ns
HD SYN vs. HD CBA	< 0.0001	****
HD SYN vs. LD CBA	0.999	ns
HD SYN vs. WT	0.9997	ns
HD SYN vs. KO	< 0.0001	****
LD SYN vs. HD CAG	> 0.9999	ns
LD SYN vs. LD CAG	0.9986	ns
LD SYN vs. HD CBA	< 0.0001	****
LD SYN vs. LD CBA	0.999	ns
LD SYN vs. WT	0.9997	ns
LD SYN vs. KO	< 0.0001	****
HD CAG vs. LD CAG	> 0.9999	ns
HD CAG vs. HD CBA	0.0002	***
HD CAG vs. LD CBA	> 0.9999	ns
HD CAG vs. WT	0.992	ns
HD CAG vs. KO	< 0.0001	****
LD CAG vs. HD CBA	0.0004	***
LD CAG vs. LD CBA	> 0.9999	ns
LD CAG vs. WT	0.947	ns
LD CAG vs. KO	< 0.0001	****
HD CBA vs. LD CBA	0.0004	***
HD CBA vs. WT	0.9949	ns
HD CBA vs. KO	< 0.0001	****
LD CBA vs. WT	< 0.0001	****
LD CBA vs. KO	0.9546	ns
WT vs. KO	< 0.0001	****

Adjusted average spleen weight

One-way ANOVA, Tukey's multiple comparison test

	P value	Summary
HD SYN vs. LD SYN	0.9997	ns
HD SYN vs. HD CAG	0.9729	ns
HD SYN vs. LD CAG	0.2921	ns
HD SYN vs. HD CBA	0.9823	ns
HD SYN vs. LD CBA	0.9745	ns
HD SYN vs. WT	>0.9999	ns
HD SYN vs. KO	>0.9999	ns
LD SYN vs. HD CAG	0.9994	ns
LD SYN vs. LD CAG	0.0893	ns
LD SYN vs. HD CBA	0.9997	ns
LD SYN vs. LD CBA	0.9995	ns
LD SYN vs. WT	0.9861	ns
LD SYN vs. KO	>0.9999	ns
HD CAG vs. LD CAG	0.0223	*
HD CAG vs. HD CBA	>0.9999	ns
HD CAG vs. LD CBA	>0.9999	ns
HD CAG vs. WT	0.852	ns
HD CAG vs. KO	0.9981	ns
LD CAG vs. HD CBA	0.0336	*
LD CAG vs. LD CBA	0.023	*
LD CAG vs. WT	0.5361	ns
LD CAG vs. KO	0.1157	ns
HD CBA vs. LD CBA	>0.9999	ns
HD CBA vs. WT	0.8879	ns
HD CBA vs. KO	0.999	ns
LD CBA vs. WT	0.8571	ns
LD CBA vs. KO	0.9983	ns
WT vs. KO	0.9936	ns

GCase activity

One-way ANOVA, Tukey's multiple comparison test

	Liver		Spleen		Lung		Heart	
	P value	Summary	P value	Summary	P value	Summary	P value	Summary
HD SYN vs. LD SYN	0.9997	ns	> 0.9999	ns	> 0.9999	ns	> 0.9999	ns
HD SYN vs. HD CAG	< 0.0001	****	< 0.0001	****	< 0.0001	****	< 0.0001	****
HD SYN vs. LD CAG	0.0002	***	0.8425	ns	0.9093	ns	< 0.0001	****
HD SYN vs. HD CBA	< 0.0001	****	0.9611	ns	0.9723	ns	< 0.0001	****
HD SYN vs. LD CBA	0.758	ns	> 0.9999	ns	> 0.9999	ns	0.9834	ns
HD SYN vs. WT	< 0.0001	****	< 0.0001	****	0.9803	ns	> 0.9999	ns
HD SYN vs. KO	0.9997	ns	0.9998	ns	> 0.9999	ns	> 0.9999	ns
LD SYN vs. HD CAG	< 0.0001	****	< 0.0001	****	< 0.0001	****	< 0.0001	****
LD SYN vs. LD CAG	< 0.0001	****	0.8321	ns	0.8788	ns	< 0.0001	****
LD SYN vs. HD CBA	< 0.0001	****	0.9567	ns	0.9595	ns	< 0.0001	****
LD SYN vs. LD CBA	0.4537	ns	> 0.9999	ns	> 0.9999	ns	0.9778	ns
LD SYN vs. WT	< 0.0001	****	< 0.0001	****	0.9704	ns	> 0.9999	ns
LD SYN vs. KO	> 0.9999	ns	0.9997	ns	> 0.9999	ns	> 0.9999	ns
HD CAG vs. LD CAG	0.0044	**	0.0036	**	< 0.0001	****	< 0.0001	****
HD CAG vs. HD CBA	0.0558	ns	0.0011	**	< 0.0001	****	0.9946	ns
HD CAG vs. LD CBA	< 0.0001	****	0.0015	**	< 0.0001	****	< 0.0001	****
HD CAG vs. WT	0.8872	ns	< 0.0001	****	< 0.0001	****	< 0.0001	****
HD CAG vs. KO	< 0.0001	****	0.0001	***	< 0.0001	****	< 0.0001	****
LD CAG vs. HD CBA	0.9862	ns	> 0.9999	ns	> 0.9999	ns	< 0.0001	****
LD CAG vs. LD CBA	0.0632	ns	0.9849	ns	0.923	ns	< 0.0001	****
LD CAG vs. WT	< 0.0001	****	< 0.0001	****	> 0.9999	ns	< 0.0001	****
LD CAG vs. KO	< 0.0001	****	0.9808	ns	0.8532	ns	< 0.0001	****
HD CBA vs. LD CBA	0.0061	**	0.9987	ns	0.9782	ns	< 0.0001	****
HD CBA vs. WT	0.0011	**	< 0.0001	****	> 0.9999	ns	< 0.0001	****
HD CBA vs. KO	< 0.0001	****	0.9988	ns	0.9445	ns	< 0.0001	****
LD CBA vs. WT	< 0.0001	****	< 0.0001	****	0.9848	ns	0.9833	ns
LD CBA vs. KO	0.4609	ns	> 0.9999	ns	> 0.9999	ns	0.974	ns
WT vs. KO	< 0.0001	****	< 0.0001	****	0.9578	ns	> 0.9999	ns

GluCer

One-way ANOVA, Tukey's multiple comparison test

	Liver		Spleen		Lung		Heart	
	P value	Summary	P value	Summary	P value	Summary	P value	Summary
HD SYN vs. LD SYN	0.0421	*	0.2449	ns	0.0172	*	>0.9999	ns
HD SYN vs. HD CAG	0.9999	ns	0.0942	ns	<0.0001	****	0.9826	ns
HD SYN vs. LD CAG	>0.9999	ns	0.6518	ns	<0.0001	****		
HD SYN vs. HD CBA	>0.9999	ns	0.0218	*	<0.0001	****	0.9636	ns
HD SYN vs. LD CBA	0.9828	ns	0.8006	ns	<0.0001	****	0.9999	ns
HD SYN vs. WT	>0.9999	ns	0.0049	**	<0.0001	****	0.9711	ns
HD SYN vs. KO	0.0002	***	>0.9999	ns	<0.0001	****	0.0155	*
LD SYN vs. HD CAG	0.0095	**	<0.0001	****	<0.0001	****	0.9904	ns
LD SYN vs. LD CAG	0.0208	*	0.0018	**	<0.0001	****		
LD SYN vs. HD CBA	0.0579	ns	<0.0001	****	<0.0001	****	0.9825	ns
LD SYN vs. LD CBA	0.4791	ns	0.005	**	<0.0001	****	0.9995	ns
LD SYN vs. WT	0.0141	*	<0.0001	****	<0.0001	****	0.9867	ns
LD SYN vs. KO	0.5507	ns	0.3039	ns	<0.0001	****	0.0152	*
HD CAG vs. LD CAG	>0.9999	ns	0.9386	ns	0.9106	ns		
HD CAG vs. HD CBA	0.9999	ns	0.999	ns	>0.9999	ns	>0.9999	ns
HD CAG vs. LD CBA	0.8843	ns	0.8818	ns	0.981	ns	0.9529	ns
HD CAG vs. WT	>0.9999	ns	0.9555	ns	>0.9999	ns	>0.9999	ns
HD CAG vs. KO	<0.0001	****	0.1094	ns	0.2091	ns	0.0407	*
LD CAG vs. HD CBA	>0.9999	ns	0.657	ns	0.9142	ns		
LD CAG vs. LD CBA	0.9569	ns	>0.9999	ns	>0.9999	ns		
LD CAG vs. WT	>0.9999	ns	0.3331	ns	0.9117	ns		
LD CAG vs. KO	<0.0001	****	0.6675	ns	0.827	ns		
HD CBA vs. LD CBA	0.9863	ns	0.5567	ns	0.9821	ns	0.9097	ns
HD CBA vs. WT	>0.9999	ns	0.9996	ns	>0.9999	ns	>0.9999	ns
HD CBA vs. KO	0.0003	***	0.0276	*	0.2128	ns	0.0045	**
LD CBA vs. WT	0.926	ns	0.2623	ns	0.9813	ns	0.9223	ns
LD CBA vs. KO	0.0112	*	0.8078	ns	0.7093	ns	0.0729	ns
WT vs. KO	<0.0001	****	0.0068	**	0.2102	ns	0.005	**

Figure 5

Average immunereactivity/pixel

One-way ANOVA, Tukey's multiple comparison test

	CD68		LAMP1	
	P value	Summary	P value	Summary
HD SYN vs. LD SYN	0.7831	ns	0.8434	ns
HD SYN vs. HD CAG	0.0914	ns	> 0.9999	ns
HD SYN vs. LD CAG	> 0.9999	ns	0.1381	ns
HD SYN vs. HD CBA	0.3652	ns	0.0884	ns
HD SYN vs. LD CBA	0.0041	**	< 0.0001	****
HD SYN vs. WT	> 0.9999	ns	> 0.9999	ns
HD SYN vs. KO	< 0.0001	****	< 0.0001	****
LD SYN vs. HD CAG	> 0.9999	ns	> 0.9999	ns
LD SYN vs. LD CAG	0.1933	ns	> 0.9999	ns
LD SYN vs. HD CBA	> 0.9999	ns	> 0.9999	ns
LD SYN vs. LD CBA	> 0.9999	ns	0.0002	***
LD SYN vs. WT	0.2804	ns	0.5368	ns
LD SYN vs. KO	0.1483	ns	< 0.0001	****
HD CAG vs. LD CAG	0.0176	*	> 0.9999	ns
HD CAG vs. HD CBA	> 0.9999	ns	> 0.9999	ns
HD CAG vs. LD CBA	> 0.9999	ns	< 0.0001	****
HD CAG vs. WT	0.0271	*	> 0.9999	ns
HD CAG vs. KO	> 0.9999	ns	< 0.0001	****
LD CAG vs. HD CBA	0.0816	ns	> 0.9999	ns
LD CAG vs. LD CBA	0.0006	***	0.002	**
LD CAG vs. WT	> 0.9999	ns	0.0813	ns
LD CAG vs. KO	< 0.0001	****	< 0.0001	****
HD CBA vs. LD CBA	> 0.9999	ns	0.0034	**
HD CBA vs. WT	0.1213	ns	0.0513	ns
HD CBA vs. KO	0.3387	ns	< 0.0001	****
LD CBA vs. WT	0.001	**	< 0.0001	****
LD CBA vs. KO	> 0.9999	ns	0.0007	***
KO vs. WT	< 0.0001	****	< 0.0001	****

Average immunereactivity/pixel CD68

One-way ANOVA, Tukey's multiple comparison test

	Spleen		Lung		Heart	
	P value	Summary	P value	Summary	P value	Summary
HD SYN vs. LD SYN	0.0575	ns	> 0.9999	ns	> 0.9999	ns
HD SYN vs. HD CAG	0.3466	ns	> 0.9999	ns	0.0536	ns
HD SYN vs. LD CAG	0.363	ns	> 0.9999	ns	> 0.9999	ns
HD SYN vs. HD CBA	0.0013	**	> 0.9999	ns	0.0005	***
HD SYN vs. LD CBA	< 0.0001	****	> 0.9999	ns	0.0037	**
HD SYN vs. WT	> 0.9999	ns	0.3243	ns	> 0.9999	ns
HD SYN vs. KO	< 0.0001	****	> 0.9999	ns	< 0.0001	****
LD SYN vs. HD CAG	> 0.9999	ns	0.691	ns	0.1996	ns
LD SYN vs. LD CAG	> 0.9999	ns	0.1557	ns	> 0.9999	ns
LD SYN vs. HD CBA	> 0.9999	ns	> 0.9999	ns	0.0023	**
LD SYN vs. LD CBA	0.199	ns	> 0.9999	ns	0.0165	*
LD SYN vs. WT	0.0002	***	0.007	**	> 0.9999	ns
LD SYN vs. KO	0.5771	ns	> 0.9999	ns	< 0.0001	****
HD CAG vs. LD CAG	> 0.9999	ns	> 0.9999	ns	> 0.9999	ns
HD CAG vs. HD CBA	> 0.9999	ns	> 0.9999	ns	> 0.9999	ns
HD CAG vs. LD CBA	0.0307	*	0.3805	ns	> 0.9999	ns
HD CAG vs. WT	0.0018	**	> 0.9999	ns	0.0078	**
HD CAG vs. KO	0.1031	ns	0.1028	ns	0.1622	ns
LD CAG vs. HD CBA	> 0.9999	ns	> 0.9999	ns	0.0503	ns
LD CAG vs. LD CBA	0.029	*	0.0791	ns	0.2726	ns
LD CAG vs. WT	0.0019	**	> 0.9999	ns	> 0.9999	ns
LD CAG vs. KO	0.098	ns	0.0184	*	0.0005	***
HD CBA vs. LD CBA	> 0.9999	ns	> 0.9999	ns	> 0.9999	ns
HD CBA vs. WT	< 0.0001	****	0.4153	ns	< 0.0001	****
HD CBA vs. KO	> 0.9999	ns	> 0.9999	ns	> 0.9999	ns
LD CBA vs. WT	< 0.0001	****	0.0032	**	0.0004	***
LD CBA vs. KO	> 0.9999	ns	> 0.9999	ns	> 0.9999	ns
KO vs. WT	< 0.0001	****	0.0006	***	< 0.0001	****

Average immunereactivity/pixel LAMP1

One-way ANOVA, Tukey's multiple comparison test

	Spleen		Lung		Heart	
	P value	Summary	P value	Summary	P value	Summary
HD SYN vs. LD SYN	> 0.9999	ns	> 0.9999	ns	> 0.9999	ns
HD SYN vs. HD CAG	> 0.9999	ns	> 0.9999	ns	0.0018	**
HD SYN vs. LD CAG	< 0.0001	****	< 0.0001	****	0.0015	**
HD SYN vs. HD CBA	< 0.0001	****	< 0.0001	****	< 0.0001	****
HD SYN vs. LD CBA	< 0.0001	****	< 0.0001	****	< 0.0001	****
HD SYN vs. WT	> 0.9999	ns	> 0.9999	ns	> 0.9999	ns
HD SYN vs. KO	< 0.0001	****	< 0.0001	****	< 0.0001	****
LD SYN vs. HD CAG	> 0.9999	ns	> 0.9999	ns	0.0026	**
LD SYN vs. LD CAG	< 0.0001	****	< 0.0001	****	0.0023	**
LD SYN vs. HD CBA	< 0.0001	****	< 0.0001	****	< 0.0001	****
LD SYN vs. LD CBA	< 0.0001	****	< 0.0001	****	< 0.0001	****
LD SYN vs. WT	> 0.9999	ns	0.2597	ns	> 0.9999	ns
LD SYN vs. KO	< 0.0001	****	< 0.0001	****	< 0.0001	****
HD CAG vs. LD CAG	< 0.0001	****	< 0.0001	****	> 0.9999	ns
HD CAG vs. HD CBA	< 0.0001	****	< 0.0001	****	> 0.9999	ns
HD CAG vs. LD CBA	< 0.0001	****	< 0.0001	****	> 0.9999	ns
HD CAG vs. WT	> 0.9999	ns	0.1587	ns	0.0024	**
HD CAG vs. KO	< 0.0001	****	< 0.0001	****	0.0033	**
LD CAG vs. HD CBA	> 0.9999	ns	> 0.9999	ns	> 0.9999	ns
LD CAG vs. LD CBA	< 0.0001	****	> 0.9999	ns	> 0.9999	ns
LD CAG vs. WT	< 0.0001	****	< 0.0001	****	0.0021	**
LD CAG vs. KO	< 0.0001	****	> 0.9999	ns	0.0037	**
HD CBA vs. LD CBA	< 0.0001	****	> 0.9999	ns	> 0.9999	ns
HD CBA vs. WT	< 0.0001	****	< 0.0001	****	< 0.0001	****
HD CBA vs. KO	< 0.0001	****	> 0.9999	ns	0.1783	ns
LD CBA vs. WT	< 0.0001	****	< 0.0001	****	< 0.0001	****
LD CBA vs. KO	> 0.9999	ns	> 0.9999	ns	0.7594	ns
KO vs. WT	< 0.0001	****	< 0.0001	****	< 0.0001	****

Supplementary 1

GCase activity vs. untransfected control

	One-way ANOVA, Tukey's multiple comparison test			
	Cell lysate		Supernatant	
	P value	Summary	P value	Summary
pAAV-hSYN-hGBA vs. untransfected control	0.0072	**	0.3161	ns
pAAV-CAG-hGBA vs. untransfected control	< 0.0001	****	< 0.0001	****
pAAV-CBA-hGBA vs. untransfected control	< 0.0001	****	< 0.0001	****

Supplementary 2

Average immunereactivity/pixel CD68

One-way ANOVA, Tukey's multiple comparison test

	VPM/VPL		Gi	
	P value	Summary	P value	Summary
HD SYN vs. LD SYN	< 0.0001	****	0.454	ns
HD SYN vs. HD CAG	> 0.9999	ns	> 0.9999	ns
HD SYN vs. LD CAG	< 0.0001	****	< 0.0001	****
HD SYN vs. HD CBA	< 0.0001	****	< 0.0001	****
HD SYN vs. LD CBA	< 0.0001	****	< 0.0001	****
HD SYN vs. WT	> 0.9999	ns	> 0.9999	ns
HD SYN vs. KO	< 0.0001	****	< 0.0001	****
LD SYN vs. HD CAG	< 0.0001	****	0.8472	ns
LD SYN vs. LD CAG	> 0.9999	ns	< 0.0001	****
LD SYN vs. HD CBA	0.0188	*	< 0.0001	****
LD SYN vs. LD CBA	> 0.9999	ns	< 0.0001	****
LD SYN vs. WT	< 0.0001	****	0.4685	ns
LD SYN vs. KO	0.2002	ns	< 0.0001	****
HD CAG vs. LD CAG	< 0.0001	****	< 0.0001	****
HD CAG vs. HD CBA	< 0.0001	****	< 0.0001	****
HD CAG vs. LD CBA	< 0.0001	****	< 0.0001	****
HD CAG vs. WT	> 0.9999	ns	> 0.9999	ns
HD CAG vs. KO	< 0.0001	****	< 0.0001	****
LD CAG vs. HD CBA	> 0.9999	ns	> 0.9999	ns
LD CAG vs. LD CBA	> 0.9999	ns	> 0.9999	ns
LD CAG vs. WT	< 0.0001	****	< 0.0001	****
LD CAG vs. KO	> 0.9999	ns	> 0.9999	ns
HD CBA vs. LD CBA	> 0.9999	ns	> 0.9999	ns
HD CBA vs. WT	< 0.0001	****	< 0.0001	****
HD CBA vs. KO	> 0.9999	ns	> 0.9999	ns
LD CBA vs. WT	< 0.0001	****	< 0.0001	****
LD CBA vs. KO	> 0.9999	ns	> 0.9999	ns
WT vs. KO	< 0.0001	****	< 0.0001	****

Average immunereactivity/pixel GFAP

One-way ANOVA, Tukey's multiple comparison test

	VPM/VPL		Gi	
	P value	Summary	P value	Summary
HD SYN vs. LD SYN	< 0.0001	****	< 0.0001	****
HD SYN vs. HD CAG	> 0.9999	ns	> 0.9999	ns
HD SYN vs. LD CAG	< 0.0001	****	< 0.0001	****
HD SYN vs. HD CBA	< 0.0001	****	< 0.0001	****
HD SYN vs. LD CBA	< 0.0001	****	< 0.0001	****
HD SYN vs. WT	> 0.9999	ns	> 0.9999	ns
HD SYN vs. KO	< 0.0001	****	< 0.0001	****
LD SYN vs. HD CAG	< 0.0001	****	< 0.0001	****
LD SYN vs. LD CAG	0.5925	ns	> 0.9999	ns
LD SYN vs. HD CBA	> 0.9999	ns	> 0.9999	ns
LD SYN vs. LD CBA	> 0.9999	ns	> 0.9999	ns
LD SYN vs. WT	< 0.0001	****	< 0.0001	****
LD SYN vs. KO	> 0.9999	ns	0.0384	*
HD CAG vs. LD CAG	< 0.0001	****	< 0.0001	****
HD CAG vs. HD CBA	< 0.0001	****	0.0005	***
HD CAG vs. LD CBA	< 0.0001	****	0.0009	***
HD CAG vs. WT	> 0.9999	ns	0.0787	ns
HD CAG vs. KO	< 0.0001	****	< 0.0001	****
LD CAG vs. HD CBA	0.5801	ns	> 0.9999	ns
LD CAG vs. LD CBA	0.2686	ns	> 0.9999	ns
LD CAG vs. WT	< 0.0001	****	< 0.0001	****
LD CAG vs. KO	0.1678	ns	0.037	*
HD CBA vs. LD CBA	> 0.9999	ns	> 0.9999	ns
HD CBA vs. WT	< 0.0001	****	< 0.0001	****
HD CBA vs. KO	> 0.9999	ns	0.0055	**
LD CBA vs. WT	< 0.0001	****	< 0.0001	****
LD CBA vs. KO	> 0.9999	ns	0.0035	**
WT vs. KO	< 0.0001	****	< 0.0001	****

Average immunereactivity/pixel LAMP1

One-way ANOVA, Tukey's multiple comparison test

	VPM/VPL		Gi	
	P value	Summary	P value	Summary
HD SYN vs. L	> 0.9999	ns	> 0.9999	ns
HD SYN vs. H	> 0.9999	ns	> 0.9999	ns
HD SYN vs. L	0.0051	**	< 0.0001	****
HD SYN vs. H	0.0002	***	< 0.0001	****
HD SYN vs. L	< 0.0001	****	< 0.0001	****
HD SYN vs. W	> 0.9999	ns	> 0.9999	ns
HD SYN vs. K	< 0.0001	****	< 0.0001	****
LD SYN vs. H	> 0.9999	ns	> 0.9999	ns
LD SYN vs. L	0.146	ns	< 0.0001	****
LD SYN vs. H	0.0058	**	< 0.0001	****
LD SYN vs. L	0.0002	***	< 0.0001	****
LD SYN vs. W	> 0.9999	ns	> 0.9999	ns
LD SYN vs. K	0.0005	***	< 0.0001	****
HD CAG vs. L	0.001	***	< 0.0001	****
HD CAG vs. H	< 0.0001	****	< 0.0001	****
HD CAG vs. L	< 0.0001	****	< 0.0001	****
HD CAG vs. W	> 0.9999	ns	> 0.9999	ns
HD CAG vs. K	< 0.0001	****	< 0.0001	****
LD CAG vs. H	> 0.9999	ns	> 0.9999	ns
LD CAG vs. L	0.3355	ns	> 0.9999	ns
LD CAG vs. W	0.0038	**	< 0.0001	****
LD CAG vs. K	0.9771	ns	> 0.9999	ns
HD CBA vs. L	> 0.9999	ns	> 0.9999	ns
HD CBA vs. W	0.0001	***	< 0.0001	****
HD CBA vs. K	> 0.9999	ns	> 0.9999	ns
LD CBA vs. W	< 0.0001	****	< 0.0001	****
LD CBA vs. K	> 0.9999	ns	> 0.9999	ns
WT vs. KO	< 0.0001	****	< 0.0001	****

Supplementary 3

GluCer

Two-way ANOVA, Tukey's multiple comparison test

	Brain		Liver		Spleen		Lung		Heart	
	P value	Summary	P value	Summary	P value	Summary	P value	Summary	P value	Summary
**C16:0**										
WT vs. KO	0.0145	*	< 0.0001	****	0.0006	***	0.1019	ns	< 0.0001	****
WT vs. HD SYN	> 0.9999	ns	0.9987	ns	0.2237	ns	< 0.0001	****	0.3826	ns
WT vs. LD SYN	0.9939	ns	< 0.0001	****	< 0.0001	****	< 0.0001	****	0.1804	ns
WT vs. HD CAG	> 0.9999	ns	> 0.9999	ns	> 0.9999	ns	> 0.9999	ns	> 0.9999	ns
WT vs. LD CAG	0.9995	ns	> 0.9999	ns	0.9558	ns	0.9572	ns	> 0.9999	ns
WT vs. HD CBA	0.9989	ns	0.9988	ns	> 0.9999	ns	> 0.9999	ns	> 0.9999	ns
WT vs. LD CBA	0.3585	ns	0.5212	ns	0.1688	ns	0.9349	ns	0.0052	**
KO vs. HD SYN	0.0224	*	< 0.0001	****	0.5741	ns	< 0.0001	****	< 0.0001	****
KO vs. LD SYN	0.1631	ns	0.0319	*	0.0213	*	< 0.0001	****	< 0.0001	****
KO vs. HD CAG	0.0152	*	< 0.0001	****	0.0017	**	0.0949	ns	< 0.0001	****
KO vs. LD CAG	0.0687	ns	< 0.0001	****	0.0281	*	0.5803	ns	< 0.0001	****
KO vs. HD CBA	0.0815	ns	< 0.0001	****	0.0007	***	0.1019	ns	< 0.0001	****
KO vs. LD CBA	0.9259	ns	< 0.0001	****	0.6577	ns	0.6789	ns	< 0.0001	****
HD SYN vs. LD SYN	0.9963	ns	< 0.0001	****	< 0.0001	****	> 0.9999	ns	> 0.9999	ns
HD SYN vs. HD CAG	> 0.9999	ns	0.997	ns	0.3595	ns	< 0.0001	****	0.4213	ns
HD SYN vs. LD CAG	0.9998	ns	0.9996	ns	0.8691	ns	< 0.0001	****	0.4682	ns
HD SYN vs. HD CBA	0.9994	ns	> 0.9999	ns	0.2272	ns	< 0.0001	****	0.6475	ns
HD SYN vs. LD CBA	0.4207	ns	0.88	ns	> 0.9999	ns	< 0.0001	****	0.6754	ns
LD SYN vs. HD CAG	0.9946	ns	< 0.0001	****	< 0.0001	****	< 0.0001	****	0.2124	ns
LD SYN vs. LD CAG	> 0.9999	ns	< 0.0001	****	< 0.0001	****	< 0.0001	****	0.2541	ns
LD SYN vs. HD CBA	> 0.9999	ns	< 0.0001	****	< 0.0001	****	< 0.0001	****	0.4096	ns
LD SYN vs. LD CBA	0.8702	ns	0.0084	**	< 0.0001	****	< 0.0001	****	0.823	ns
HD CAG vs. LD CAG	0.9996	ns	> 0.9999	ns	0.9904	ns	0.9498	ns	> 0.9999	ns
HD CAG vs. HD CBA	0.9991	ns	0.9971	ns	> 0.9999	ns	> 0.9999	ns	> 0.9999	ns
HD CAG vs. LD CBA	0.3671	ns	0.4665	ns	0.2846	ns	0.9255	ns	0.0072	**
LD CAG vs. HD CBA	> 0.9999	ns	0.9996	ns	0.9574	ns	0.9572	ns	> 0.9999	ns
LD CAG vs. LD CBA	0.7	ns	0.5858	ns	0.8053	ns	> 0.9999	ns	0.0104	*
HD CBA vs. LD CBA	0.74	ns	0.902	ns	0.1717	ns	0.9349	ns	0.0233	*
**C18:0**										
WT vs. KO	< 0.0001	****	0.9898	ns	0.9883	ns	> 0.9999	ns	0.0072	**
WT vs. HD SYN	> 0.9999	ns	> 0.9999	ns	0.9978	ns	0.9898	ns	> 0.9999	ns
WT vs. LD SYN	< 0.0001	****	0.9998	ns	0.7831	ns	0.9392	ns	> 0.9999	ns
WT vs. HD CAG	> 0.9999	ns	> 0.9999	ns	> 0.9999	ns	> 0.9999	ns	> 0.9999	ns
WT vs. LD CAG	< 0.0001	****	> 0.9999	ns	> 0.9999	ns	> 0.9999	ns	> 0.9999	ns
WT vs. HD CBA	< 0.0001	****	> 0.9999	ns	> 0.9999	ns	> 0.9999	ns	> 0.9999	ns
WT vs. LD CBA	< 0.0001	****	> 0.9999	ns	0.9997	ns	> 0.9999	ns	0.9988	ns
KO vs. HD SYN	< 0.0001	****	0.9935	ns	> 0.9999	ns	> 0.9999	ns	0.0243	*
KO vs. LD SYN	< 0.0001	****	> 0.9999	ns	0.9992	ns	0.9988	ns	0.02	*
KO vs. HD CAG	< 0.0001	****	0.9892	ns	0.9923	ns	> 0.9999	ns	0.0064	**
KO vs. LD CAG	< 0.0001	****	0.9913	ns	0.9979	ns	> 0.9999	ns	0.009	**
KO vs. HD CBA	< 0.0001	****	0.9969	ns	0.9866	ns	> 0.9999	ns	0.011	*
KO vs. LD CBA	< 0.0001	****	0.9997	ns	> 0.9999	ns	> 0.9999	ns	0.088	ns
HD SYN vs. LD SYN	< 0.0001	****	> 0.9999	ns	0.9911	ns	> 0.9999	ns	> 0.9999	ns
HD SYN vs. HD CAG	> 0.9999	ns	> 0.9999	ns	0.9988	ns	0.9893	ns	0.9998	ns
HD SYN vs. LD CAG	< 0.0001	****	> 0.9999	ns	0.9998	ns	0.9944	ns	0.9999	ns
HD SYN vs. HD CBA	< 0.0001	****	> 0.9999	ns	0.9974	ns	0.989	ns	> 0.9999	ns
HD SYN vs. LD CBA	< 0.0001	****	> 0.9999	ns	> 0.9999	ns	0.9974	ns	> 0.9999	ns
LD SYN vs. HD CAG	< 0.0001	****	0.9998	ns	0.8172	ns	0.9374	ns	0.9997	ns
LD SYN vs. LD CAG	> 0.9999	ns	0.9999	ns	0.892	ns	0.9583	ns	0.9998	ns
LD SYN vs. HD CBA	0.9564	ns	> 0.9999	ns	0.7712	ns	0.9364	ns	> 0.9999	ns
LD SYN vs. LD CBA	< 0.0001	****	> 0.9999	ns	0.9746	ns	0.9752	ns	> 0.9999	ns
HD CAG vs. LD CAG	< 0.0001	****	> 0.9999	ns	> 0.9999	ns	> 0.9999	ns	> 0.9999	ns
HD CAG vs. HD CBA	< 0.0001	****	> 0.9999	ns	> 0.9999	ns	> 0.9999	ns	> 0.9999	ns
HD CAG vs. LD CBA	< 0.0001	****	> 0.9999	ns	0.9999	ns	> 0.9999	ns	0.9968	ns
LD CAG vs. HD CBA	0.9865	ns	> 0.9999	ns	> 0.9999	ns	> 0.9999	ns	> 0.9999	ns
LD CAG vs. LD CBA	< 0.0001	****	> 0.9999	ns	> 0.9999	ns	> 0.9999	ns	0.9974	ns
HD CBA vs. LD CBA	< 0.0001	****	> 0.9999	ns	0.9996	ns	> 0.9999	ns	0.9985	ns
**C20:0**										
WT vs. KO	0.2977	ns	> 0.9999	ns	> 0.9999	ns	> 0.9999	ns	0.2807	ns
WT vs. HD SYN	> 0.9999	ns	> 0.9999	ns	0.9997	ns	0.9993	ns	0.8703	ns
WT vs. LD SYN	0.9653	ns	0.9969	ns	0.9724	ns	0.9952	ns	0.3832	ns
WT vs. HD CAG	> 0.9999	ns	> 0.9999	ns	> 0.9999	ns	> 0.9999	ns	> 0.9999	ns
WT vs. LD CAG	0.782	ns	> 0.9999	ns	> 0.9999	ns	> 0.9999	ns	> 0.9999	ns
WT vs. HD CBA	0.7623	ns	> 0.9999	ns	> 0.9999	ns	> 0.9999	ns	> 0.9999	ns
WT vs. LD CBA	0.4768	ns	> 0.9999	ns	> 0.9999	ns	> 0.9999	ns	0.9998	ns
KO vs. HD SYN	0.3352	ns	> 0.9999	ns	> 0.9999	ns	> 0.9999	ns	0.9538	ns
KO vs. LD SYN	0.9294	ns	> 0.9999	ns	0.9982	ns	> 0.9999	ns	0.9996	ns
KO vs. HD CAG	0.3064	ns	> 0.9999	ns	> 0.9999	ns	> 0.9999	ns	0.1906	ns
KO vs. LD CAG	0.9931	ns	> 0.9999	ns	> 0.9999	ns	> 0.9999	ns	0.2194	ns
KO vs. HD CBA	0.9947	ns	> 0.9999	ns	> 0.9999	ns	> 0.9999	ns	0.2194	ns
KO vs. LD CBA	> 0.9999	ns	> 0.9999	ns	> 0.9999	ns	> 0.9999	ns	0.6491	ns
HD SYN vs. LD SYN	0.9706	ns	0.9987	ns	0.9997	ns	> 0.9999	ns	0.9964	ns
HD SYN vs. HD CAG	> 0.9999	ns	> 0.9999	ns	> 0.9999	ns	0.9992	ns	0.7465	ns
HD SYN vs. LD CAG	0.8081	ns	> 0.9999	ns	> 0.9999	ns	0.9996	ns	0.7785	ns
HD SYN vs. HD CBA	0.79	ns	> 0.9999	ns	0.9998	ns	0.9992	ns	0.7785	ns
HD SYN vs. LD CBA	0.5161	ns	> 0.9999	ns	> 0.9999	ns	0.9998	ns	0.9932	ns
LD SYN vs. HD CAG	0.9681	ns	0.9964	ns	0.9825	ns	0.9949	ns	0.2589	ns
LD SYN vs. LD CAG	0.9998	ns	0.9971	ns	0.987	ns	0.9967	ns	0.304	ns
LD SYN vs. HD CBA	0.9997	ns	0.9987	ns	0.9765	ns	0.9948	ns	0.304	ns
LD SYN vs. LD CBA	0.9832	ns	0.9988	ns	0.9922	ns	0.9981	ns	0.8178	ns
HD CAG vs. LD CAG	0.7911	ns	> 0.9999	ns	> 0.9999	ns	> 0.9999	ns	> 0.9999	ns
HD CAG vs. HD CBA	0.7718	ns	> 0.9999	ns	> 0.9999	ns	> 0.9999	ns	> 0.9999	ns
HD CAG vs. LD CBA	0.4874	ns	> 0.9999	ns	> 0.9999	ns	> 0.9999	ns	0.9972	ns
LD CAG vs. HD CBA	> 0.9999	ns	> 0.9999	ns	> 0.9999	ns	> 0.9999	ns	> 0.9999	ns
LD CAG vs. LD CBA	0.9996	ns	> 0.9999	ns	> 0.9999	ns	> 0.9999	ns	0.9977	ns
HD CBA vs. LD CBA	0.9997	ns	> 0.9999	ns	> 0.9999	ns	> 0.9999	ns	0.9977	ns

**C22:0**										
WT vs. KO	0.9969	ns	0.5661	ns	0.939	ns	0.9162	ns	< 0.0001	****
WT vs. HD SYN	> 0.9999	ns	> 0.9999	ns	0.8119	ns	0.0027	**	0.5071	ns
WT vs. LD SYN	> 0.9999	ns	0.1624	ns	0.008	**	< 0.0001	****	0.6319	ns
WT vs. HD CAG	> 0.9999	ns	> 0.9999	ns	> 0.9999	ns	> 0.9999	ns	0.9998	ns
WT vs. LD CAG	> 0.9999	ns	> 0.9999	ns	0.9993	ns	> 0.9999	ns	0.9999	ns
WT vs. HD CBA	> 0.9999	ns	> 0.9999	ns	> 0.9999	ns	> 0.9999	ns	0.9999	ns
WT vs. LD CBA	0.9996	ns	> 0.9999	ns	0.9987	ns	0.9998	ns	> 0.9999	ns
KO vs. HD SYN	0.9978	ns	0.7456	ns	> 0.9999	ns	0.404	ns	0.0453	*
KO vs. LD SYN	0.9998	ns	0.9984	ns	0.3459	ns	0.0417	*	0.0196	*
KO vs. HD CAG	0.9971	ns	0.4768	ns	0.9843	ns	0.9145	ns	< 0.0001	****
KO vs. LD CAG	0.9996	ns	0.6195	ns	0.9981	ns	0.9754	ns	< 0.0001	****
KO vs. HD CBA	0.9998	ns	0.7421	ns	0.9483	ns	0.9157	ns	< 0.0001	****
KO vs. LD CBA	> 0.9999	ns	0.7634	ns	0.9992	ns	0.9912	ns	0.0009	***
HD SYN vs. LD SYN	> 0.9999	ns	0.3024	ns	0.4687	ns	0.9496	ns	> 0.9999	ns
HD SYN vs. HD CAG	> 0.9999	ns	> 0.9999	ns	0.9238	ns	0.0026	**	0.2736	ns
HD SYN vs. LD CAG	> 0.9999	ns	> 0.9999	ns	0.9807	ns	0.0079	**	0.317	ns
HD SYN vs. HD CBA	> 0.9999	ns	> 0.9999	ns	0.8314	ns	0.0026	**	0.317	ns
HD SYN vs. LD CBA	0.9997	ns	> 0.9999	ns	0.9896	ns	0.0191	*	0.8255	ns
LD SYN vs. HD CAG	> 0.9999	ns	0.1182	ns	0.0201	*	< 0.0001	****	0.3691	ns
LD SYN vs. LD CAG	> 0.9999	ns	0.1939	ns	0.0479	*	< 0.0001	****	0.4183	ns
LD SYN vs. HD CBA	> 0.9999	ns	0.3137	ns	0.0092	**	< 0.0001	****	0.4183	ns
LD SYN vs. LD CBA	> 0.9999	ns	0.3528	ns	0.0751	ns	0.0003	***	0.9042	ns
HD CAG vs. LD CAG	> 0.9999	ns	> 0.9999	ns	> 0.9999	ns	> 0.9999	ns	> 0.9999	ns
HD CAG vs. HD CBA	> 0.9999	ns	> 0.9999	ns	> 0.9999	ns	> 0.9999	ns	> 0.9999	ns
HD CAG vs. LD CBA	0.9996	ns	> 0.9999	ns	> 0.9999	ns	0.9998	ns	0.9968	ns
LD CAG vs. HD CBA	> 0.9999	ns	> 0.9999	ns	0.9996	ns	> 0.9999	ns	> 0.9999	ns
LD CAG vs. LD CBA	> 0.9999	ns	> 0.9999	ns	> 0.9999	ns	> 0.9999	ns	0.9974	ns
HD CBA vs. LD CBA	> 0.9999	ns	> 0.9999	ns	0.9992	ns	0.9998	ns	0.9974	ns
**C23:0**										
WT vs. KO	> 0.9999	ns	0.9651	ns	> 0.9999	ns	> 0.9999	ns	> 0.9999	ns
WT vs. HD SYN	> 0.9999	ns	> 0.9999	ns	0.997	ns	0.8364	ns	> 0.9999	ns
WT vs. LD SYN	> 0.9999	ns	0.0627	ns	0.5176	ns	0.6436	ns	> 0.9999	ns
WT vs. HD CAG	> 0.9999	ns	> 0.9999	ns	> 0.9999	ns	> 0.9999	ns	> 0.9999	ns
WT vs. LD CAG	> 0.9999	ns	> 0.9999	ns	> 0.9999	ns	> 0.9999	ns	> 0.9999	ns
WT vs. HD CBA	> 0.9999	ns	> 0.9999	ns	> 0.9999	ns	> 0.9999	ns	> 0.9999	ns
WT vs. LD CBA	> 0.9999	ns	> 0.9999	ns	> 0.9999	ns	> 0.9999	ns	> 0.9999	ns
KO vs. HD SYN	> 0.9999	ns	0.9852	ns	> 0.9999	ns	0.9672	ns	> 0.9999	ns
KO vs. LD SYN	> 0.9999	ns	0.604	ns	0.7879	ns	0.8909	ns	> 0.9999	ns
KO vs. HD CAG	> 0.9999	ns	0.9597	ns	> 0.9999	ns	> 0.9999	ns	> 0.9999	ns
KO vs. LD CAG	> 0.9999	ns	0.9727	ns	> 0.9999	ns	> 0.9999	ns	> 0.9999	ns
KO vs. HD CBA	> 0.9999	ns	0.9869	ns	> 0.9999	ns	> 0.9999	ns	> 0.9999	ns
KO vs. LD CBA	> 0.9999	ns	0.9956	ns	> 0.9999	ns	> 0.9999	ns	> 0.9999	ns
HD SYN vs. LD SYN	> 0.9999	ns	0.1093	ns	0.9338	ns	> 0.9999	ns	> 0.9999	ns
HD SYN vs. HD CAG	> 0.9999	ns	> 0.9999	ns	0.9994	ns	0.8341	ns	> 0.9999	ns
HD SYN vs. LD CAG	> 0.9999	ns	> 0.9999	ns	0.9998	ns	0.8847	ns	> 0.9999	ns
HD SYN vs. HD CBA	> 0.9999	ns	> 0.9999	ns	0.9975	ns	0.8325	ns	> 0.9999	ns
HD SYN vs. LD CBA	> 0.9999	ns	> 0.9999	ns	0.9996	ns	0.8919	ns	> 0.9999	ns
LD SYN vs. HD CAG	> 0.9999	ns	0.0578	ns	0.6266	ns	0.6405	ns	> 0.9999	ns
LD SYN vs. LD CAG	> 0.9999	ns	0.0714	ns	0.684	ns	0.7142	ns	> 0.9999	ns
LD SYN vs. HD CBA	> 0.9999	ns	0.1348	ns	0.5319	ns	0.6382	ns	> 0.9999	ns
LD SYN vs. LD CBA	> 0.9999	ns	0.2244	ns	0.6793	ns	0.7313	ns	> 0.9999	ns
HD CAG vs. LD CAG	> 0.9999	ns	> 0.9999	ns	> 0.9999	ns	> 0.9999	ns	> 0.9999	ns
HD CAG vs. HD CBA	> 0.9999	ns	> 0.9999	ns	> 0.9999	ns	> 0.9999	ns	> 0.9999	ns
HD CAG vs. LD CBA	> 0.9999	ns	> 0.9999	ns	> 0.9999	ns	> 0.9999	ns	> 0.9999	ns
LD CAG vs. HD CBA	> 0.9999	ns	> 0.9999	ns	> 0.9999	ns	> 0.9999	ns	> 0.9999	ns
LD CAG vs. LD CBA	> 0.9999	ns	> 0.9999	ns	> 0.9999	ns	> 0.9999	ns	> 0.9999	ns
HD CBA vs. LD CBA	> 0.9999	ns	> 0.9999	ns	> 0.9999	ns	> 0.9999	ns	> 0.9999	ns
**C24:0**										
WT vs. KO	> 0.9999	ns	0.2525	ns	0.4879	ns	0.0071	**	< 0.0001	****
WT vs. HD SYN	> 0.9999	ns	> 0.9999	ns	0.452	ns	< 0.0001	****	0.8127	ns
WT vs. LD SYN	> 0.9999	ns	0.756	ns	< 0.0001	****	< 0.0001	****	0.9797	ns
WT vs. HD CAG	> 0.9999	ns	> 0.9999	ns	0.9992	ns	> 0.9999	ns	> 0.9999	ns
WT vs. LD CAG	> 0.9999	ns	> 0.9999	ns	0.9396	ns	0.6048	ns	0.9991	ns
WT vs. HD CBA	> 0.9999	ns	> 0.9999	ns	> 0.9999	ns	> 0.9999	ns	> 0.9999	ns
WT vs. LD CBA	> 0.9999	ns	0.9995	ns	0.9186	ns	0.8753	ns	0.8831	ns
KO vs. HD SYN	> 0.9999	ns	0.3583	ns	> 0.9999	ns	< 0.0001	****	0.012	*
KO vs. LD SYN	> 0.9999	ns	0.9906	ns	0.0141	*	< 0.0001	****	0.0015	**
KO vs. HD CAG	> 0.9999	ns	0.2252	ns	0.8268	ns	0.007	**	< 0.0001	****
KO vs. LD CAG	> 0.9999	ns	0.2825	ns	0.9862	ns	0.4062	ns	< 0.0001	****
KO vs. HD CBA	> 0.9999	ns	0.4072	ns	0.6577	ns	0.0073	**	0.0002	***
KO vs. LD CBA	> 0.9999	ns	0.7349	ns	0.9943	ns	0.2346	ns	0.0214	*
HD SYN vs. LD SYN	> 0.9999	ns	0.8496	ns	0.0084	**	< 0.0001	****	0.9993	ns
HD SYN vs. HD CAG	> 0.9999	ns	> 0.9999	ns	0.8093	ns	< 0.0001	****	0.5987	ns
HD SYN vs. LD CAG	> 0.9999	ns	> 0.9999	ns	0.985	ns	< 0.0001	****	0.5071	ns
HD SYN vs. HD CBA	> 0.9999	ns	> 0.9999	ns	0.628	ns	< 0.0001	****	0.9095	ns
HD SYN vs. LD CBA	> 0.9999	ns	> 0.9999	ns	0.994	ns	< 0.0001	****	> 0.9999	ns
LD SYN vs. HD CAG	> 0.9999	ns	0.7201	ns	< 0.0001	****	< 0.0001	****	0.8884	ns
LD SYN vs. LD CAG	> 0.9999	ns	0.7906	ns	0.0001	***	< 0.0001	****	0.818	ns
LD SYN vs. HD CBA	> 0.9999	ns	0.8741	ns	< 0.0001	****	< 0.0001	****	0.9947	ns
LD SYN vs. LD CBA	> 0.9999	ns	0.9869	ns	0.0004	***	< 0.0001	****	0.9997	ns
HD CAG vs. LD CAG	> 0.9999	ns	> 0.9999	ns	0.9988	ns	0.6017	ns	> 0.9999	ns
HD CAG vs. HD CBA	> 0.9999	ns	> 0.9999	ns	> 0.9999	ns	> 0.9999	ns	0.9997	ns
HD CAG vs. LD CBA	> 0.9999	ns	0.999	ns	0.9972	ns	0.8734	ns	0.711	ns
LD CAG vs. HD CBA	> 0.9999	ns	> 0.9999	ns	0.9854	ns	0.6122	ns	0.9976	ns
LD CAG vs. LD CBA	> 0.9999	ns	0.9997	ns	> 0.9999	ns	> 0.9999	ns	0.6232	ns
HD CBA vs. LD CBA	> 0.9999	ns	> 0.9999	ns	0.9764	ns	0.8797	ns	0.9453	ns

Supplementary 5

VCN

One-way ANOVA, Tukey's multiple comparison test

	Brain		Liver		Spleen		Lung		Heart		Bone marrow	
	P value	Summary	P value	Summary	P value	Summary	P value	Summary	P value	Summary	P value	Summary
HD SYN vs. LD SYN	0.0007	***	0.0058	**	0.743	ns	0.0038	**	0.0038	**	0.8182	ns
HD SYN vs. HD CAG	0.9349	ns	0.3665	ns	0.9084	ns	0.1167	ns	0.1167	ns	0.7398	ns
HD SYN vs. LD CAG	0.0009	***	0.0033	**	> 0.9999	ns	0.0137	*	0.0137	*	0.9991	ns
HD SYN vs. HD CBA	0.9849	ns	0.5357	ns	0.094	ns	0.0675	ns	0.0675	ns	> 0.9999	ns
HD SYN vs. LD CBA	0.0007	***	0.0606	ns	> 0.9999	ns	0.0195	*	0.0195	*		
LD SYN vs. HD CAG	0.0081	**	0.4493	ns	0.9991	ns	< 0.0001	****	< 0.0001	****	> 0.9999	ns
LD SYN vs. LD CAG	> 0.9999	ns	> 0.9999	ns	0.6318	ns	0.9973	ns	0.9973	ns	0.978	ns
LD SYN vs. HD CBA	0.0036	**	< 0.0001	****	0.7362	ns	< 0.0001	****	< 0.0001	****	0.9369	ns
LD SYN vs. LD CBA	> 0.9999	ns	0.9669	ns	0.8251	ns	0.9968	ns	0.9968	ns		
HD CAG vs. LD CAG	0.0105	*	0.3383	ns	0.8327	ns	< 0.0001	****	< 0.0001	****	0.9554	ns
HD CAG vs. HD CBA	0.9998	ns	0.0053	**	0.5104	ns	0.9999	ns	0.9999	ns	0.8951	ns
HD CAG vs. LD CBA	0.0077	**	0.918	ns	0.9351	ns	< 0.0001	****	< 0.0001	****		
LD CAG vs. HD CBA	0.0047	**	< 0.0001	****	0.0621	ns	< 0.0001	****	< 0.0001	****	> 0.9999	ns
LD CAG vs. LD CBA	> 0.9999	ns	0.921	ns	> 0.9999	ns	> 0.9999	ns	> 0.9999	ns		
HD CBA vs. LD CBA	0.0035	**	0.0003	***	0.2156	ns	< 0.0001	****	< 0.0001	****		
