# Supplementary material for: Comparison of different promoters to improve AAV vector-mediated gene therapy for neuronopathic Gaucher disease
Source: Hum Mol Genet. 2024 May 16;33(17):1467–80. doi: 10.1093/hmg/ddae081 (PMC11336133; doi:10.1093/hmg/ddae081)
Supplement: Supplementary_legends_ddae081 [file supplementary_legends_ddae081.docx]

**Figure S1 – *In vitro* testing. A** Schematics of the plasmid maps. **B** Quantification of GCase activity in cell lysate following plasmid transfection. **C** Quantification of secreted GCase activity in cell media following plasmid transfection.

* indicates statistically significant difference between the experimental group and WT controls. *p* values and statistical tests are reported in Table S8.

**Figure S2 – Gene therapy ameliorates the neuropathology of the nGD model.** **A** Representative images of brain sections (thalamic region VPM/VPL) stained for the macrophagic marker CD68. Scale bar: 100μm; high magnification inserts: 60μm. **B** Representative images of brain sections (brain stem region Gi) stained for CD68. Scale bar: 100μm; high magnification inserts: 60μm. **C** Representative images of brain sections (VPM/VPL) stained for the astrocytic marker GFAP. Scale bar: 100μm; high magnification inserts: 60μm. **D** Representative images of brain sections (Gi) stained for GFAP. Scale bar: 100μm; high magnification inserts: 60μm. **E** Representative images of brain sections (VPM/VPL) stained for the lysosomal marker LAMP1. Scale bar: 100μm; high magnification inserts: 60μm. **F** Representative images of brain sections (Gi) stained for LAMP1. Scale bar: 100μm; high magnification inserts: 60μm. **G** Quantification of immunoreactivity of the VPM/VPL region stained for CD68. **H** Quantification of immunoreactivity of the Gi region stained for CD68. **I** Quantification of immunoreactivity of the VPM/VPL region stained for GFAP. **J** Quantification of immunoreactivity of the Gi region stained for GFAP. **K** Quantification of immunoreactivity of the VPM/VPL region stained for LAMP1. **L** Quantification of immunoreactivity of the Gi region stained for LAMP1.

All data are presented as single data points and mean. * indicates statistically significant difference between the experimental group and WT controls; # indicates statistically significant difference between the experimental group and untreated KO controls. *p* values and statistical tests are reported in Table S9.

**Figure S3 – Evaluation of GluCer accumulation via mass spectrometry. A** Brain. **B** Liver. **C** Spleen. **D** Lung. **E** Heart.

All data are presented as average value and SD. *p* values and statistical tests are reported in Table S10 and S10.1.

**Figure 4 - Histopathological analysis of visceral organs following gene therapy. A** Representative images of spleen sections stained for the macrophagic marker CD68. Scale bar: 100μm; high magnification inserts: 60μm. **B** Representative images of lung sections stained for CD68. Scale bar: 100μm; high magnification inserts: 60μm. **C** Representative images of heart sections stained for CD68. Scale bar: 100μm; high magnification inserts: 60μm. **D** Representative images of spleen sections stained for the lysosomal marker LAMP1. Scale bar: 100μm; high magnification inserts: 60μm. **E** Representative images of lung sections stained for LAMP1. Scale bar: 100μm; high magnification inserts: 60μm. **F** Representative images of heart sections stained for LAMP1. Scale bar: 100μm; high magnification inserts: 60μm. **G** Representative images of spleen sections stained with H&E. Arrows represents Gaucher cells. Scale bar: 100μm; high magnification inserts: 60μm. **H** Representative images of lung sections stained with H&E. Arrows represents Gaucher cells. Scale bar: 100μm; high magnification inserts: 60μm. **I** Representative images of heart sections stained with H&E. Scale bar: 100μm; high magnification inserts: 60μm.

**Figure S5 – Vector copy number. A** Vector copy number analysis in brain, **B** liver, **C** spleen, **D** lung, **E** heart and F bone marrow, expressed as VCN/diploid genome.

All data are presented as single data points and mean. *p* values and statistical tests are reported in Table S11.

**Tables**

**Table S1 – Sex distribution in treatment cohorts.**

**Table S2 – Randomization of treatments.**

**Table S3 – *p* values and statistical tests for Fig. 1.**

**Table S4 – *p* values and statistical tests for Fig. 2.**

**Table S5 – *p* values and statistical tests for Fig. 3.**

**Table S6 – *p* values and statistical tests for Fig. 4.**

**Table S7 – *p* values and statistical tests for Fig. 5.**

**Table S8 – *p* values and statistical tests for Fig. S1.**

**Table S9 – *p* values and statistical tests for Fig. S2**

**Table S10 – *p* values and statistical tests for Fig. S3.**

**Table S10.1 – Continuation of Table S10.**

**Table S11 – *p* values and statistical tests for Fig. S5.**
